# GroEL protein of the *Leptospira* spp. interacts with host proteins and induces cytokines secretion on macrophages

**DOI:** 10.1186/s12866-021-02162-w

**Published:** 2021-03-31

**Authors:** Joana Dias Ho, Luiz Eduardo Massao Takara, Denize Monaris, Aline Patrícia Gonçalves, Antonio Francisco Souza-Filho, Gisele Oliveira de Souza, Marcos Bryan Heinemann, Paulo Lee Ho, Patrícia Antonia Estima Abreu

**Affiliations:** 1 Laboratory of Bacteriology, Butantan Institute, São Paulo, Brazil; 2 Laboratory of Bacterial Zoonosis, School of Veterinary Medicine and Animal Science, University of São Paulo, São Paulo, Brazil; 3 Bioindustrial Division, Butantan Institute, São Paulo, Brazil

**Keywords:** *Leptospira*, Leptospirosis, Chaperonin 60, GroEL, HSP60, J774.1 cells, Moonlighting protein

## Abstract

**Background:**

Leptospirosis is a zoonotic disease caused by infection with spirochetes from *Leptospira* genus. It has been classified into at least 17 pathogenic species, with more than 250 serologic variants. This wide distribution may be a result of leptospiral ability to colonize the renal tubules of mammalian hosts, including humans, wildlife, and many domesticated animals. Previous studies showed that the expression of proteins belonging to the microbial heat shock protein (HSP) family is upregulated during infection and also during various stress stimuli. Several proteins of this family are known to have important roles in the infectious processes in other bacteria, but the role of HSPs in *Leptospira* spp. is poorly understood. In this study, we have evaluated the capacity of the protein GroEL, a member of HSP family, of interacting with host proteins and of stimulating the production of cytokines by macrophages.

**Results:**

The binding experiments demonstrated that the recombinant GroEL protein showed interaction with several host components in a dose-dependent manner. It was also observed that GroEL is a surface protein, and it is secreted extracellularly. Moreover, two cytokines (tumor necrosis factor-α and interleukin-6) were produced when macrophages cells were stimulated with this protein.

**Conclusions:**

Our findings showed that GroEL protein may contribute to the adhesion of leptospires to host tissues and stimulate the production of proinflammatory cytokines during infection. These features might indicate an important role of GroEL in the pathogen-host interaction in the leptospirosis.

**Supplementary Information:**

The online version contains supplementary material available at 10.1186/s12866-021-02162-w.

## Background

Pathogenic spirochetes of the genus *Leptospira* are highly invasive bacteria capable of infecting human or animals and causing leptospirosis, probably the most spread zoonotic disease in the world [1, 2]. It has been estimated that 1.03 million cases of human leptospirosis occur worldwide annually with approximately 58,900 deaths [3, 4]. Transmission involves the direct contact with infected animals or indirect contact with contaminated water or soil [1, 2]. To date, 64 species of *Leptospira* are known and among them, 17 pathogenic, 21 intermediately pathogenic, and 26 non-pathogenic species have been identified, with more than 250 serologic variants described [5]. This wide distribution may be the result of their ability to survive in water or wet soil and to colonize the renal tubules of carrier hosts, allowing the maintenance of leptospires in the environment [6].

The mechanisms by which leptospires invade the host and cause the disease are not fully understood. Experimental results have shown that the pathogenesis is initiated by the ability of these bacteria to attach to extracellular matrix components (EMC) [7–13], to escape host’s immune responses [14–19], to disseminate, and to persist in the host [2, 20].

GroEL, also known as chaperonin 60 (Cpn60) or HSP60, is a member of the heat shock protein (HSP) family. HSPs constitute a highly conserved group of proteins in prokaryotes and eukaryotes, which are classified into several families, according to their molecular masses, but not by their functions [21, 22]. These proteins are expressed constitutively in the cells and can be induced by stressful situations such as elevated temperature, oxygen limitation, extreme pH, or lack of nutrients. Under these conditions, HSPs allow the cell to recover from protein denaturation, preventing improper aggregation and promoting adequate folding of the proteins. Otherwise, proteins might be denatured and degraded irreversibly. Thus, HSPs can act as molecular chaperones and/or proteases [23]. Besides these functions, these proteins can present multiple unexpected biological activities [21–23]. Some are potent immunomodulatory proteins, being able to stimulate the production of nitric oxide and several cytokines, such as tumor necrosis factor-α (TNF-α), interferon-γ (IFNγ), and interleukins (IL) IL-1β, IL-6, IL10, and IL-12 in monocytes, macrophages, and dendritic cells [24–28]. In addition, previous studies showed that the bacterial HSPs are capable of binding to host components, inducing the aggregation and promoting the biofilm formation [29–31].

In this study, we evaluated the capacity of GroEL protein of the *Leptospira* spp. to interact with host molecules and to stimulate the production of cytokines by cultured macrophages. Our results demonstrated that this protein might contribute to the adhesion of leptospires to host tissues and stimulate the production of cytokines during infection. We also report that GroEL is secreted extracellularly and exposed at the surface of the leptospires.

## Results

### In silico analysis of *L.interrogans* GroEL

**Fig. 1:**
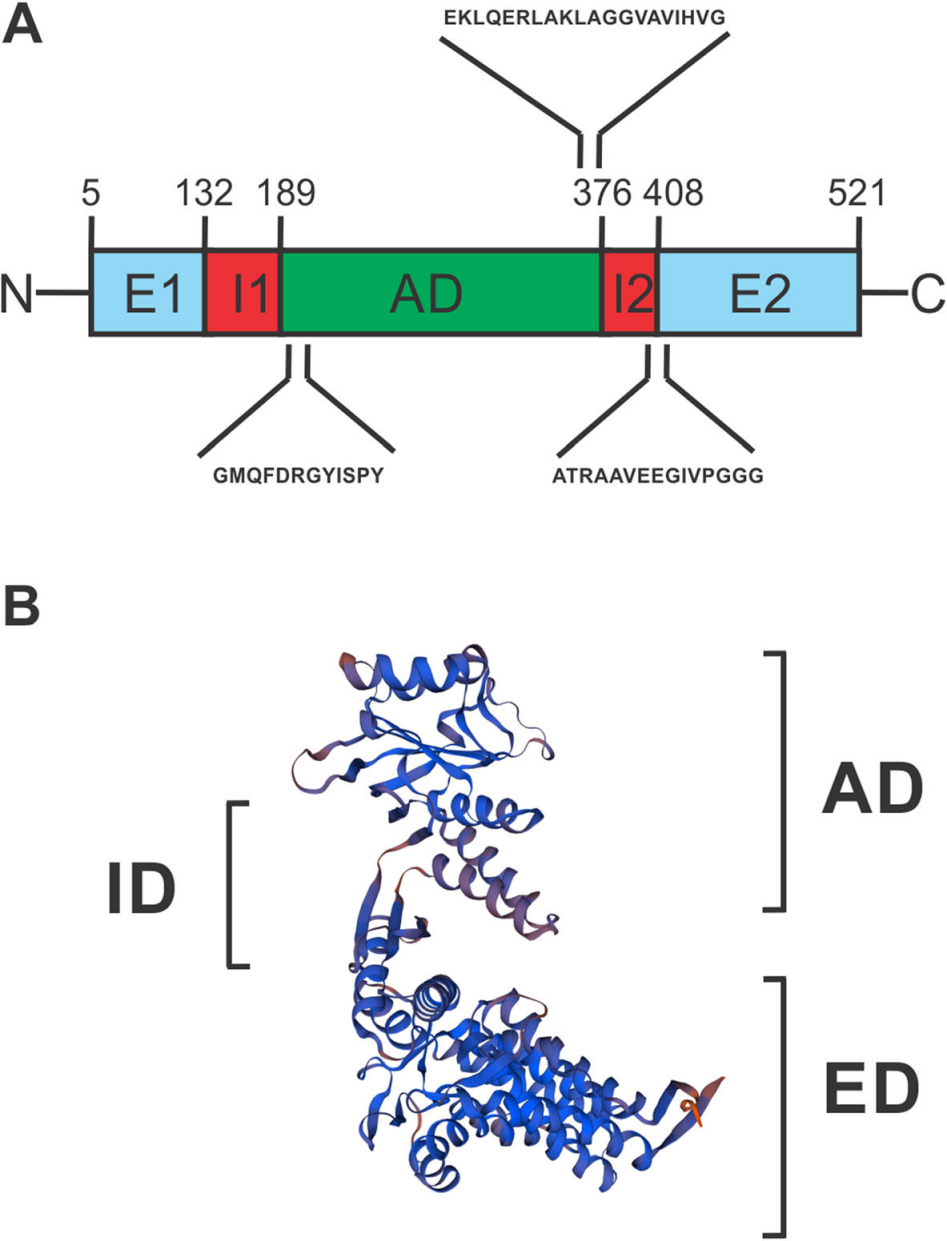
**a** Schematic representation of GroEL from *L. interrogans.* Three structural domains are presented: apical (AD), intermediate (ID) with two subdomains (I1 and I2), and equatorial (ED) consisting of subdomains E1 and E2. In detail are shown three highly conserved GroEL regions: residues 191–202 (GMQFDRGYISPY); residues 362–381 (EKLQERLAKLAGGVAVIHVG), and residues 401–415 (ATRAAVEEGIVPGGG. **b** The predicted 3D structural model of *L. interrogans* GroEL generated using the SWISS-MODEL protein homology modeling server

Conserved Domain Database (CDD) web server was used to identify putative conserved domains in *L. interrogans* GroEL. As illustrated in Fig. 1, GroEL shares a basic chaperonin structure that consists of three domains: an apical domain (AD) implicated in substrate binding; an equatorial ATP-binding (ED); an intermediate domain (ID) that connects the equatorial domain and the apical domain [32]. The equatorial domain is composed by two subdomains named E1 (residues 5–132) and E2 (residues 408–521) located at N- and C-termini of the sequence. Likewise, the intermediate domain consists of two subdomains: I1 (residues 133–189) and I2 (residues 376–408), situated on both sides of the protein. Meanwhile, the apical domain (residues 190–375) is inserted into the intermediate domain. Three others highly conserved GroEL motifs were also identified: residues 191–202 (GMQFDRGYISPY); residues 362–381 (EKLQERLAKLAGGVAVIHVG), and residues 401–415 (ATRAAVEEGIVPGGG). 3D structure prediction was also performed using SWISS-MODEL protein homology modeling server. As a result, three 3D models were generated. The crystal structure of the Cpn60 from *T. thermophilus* [33] was chosen as the best template for leptospiral GroEL modelling due the high similarity (68.18%) of the amino acid sequence in both proteins and due to reliability of GMQE (Global Model Quality Estimation) [34] and the QMEAN (Qualitative Model Energy ANalysis) scores obtained [35]. The GMQE and QMEAN were 0.81 and − 0.09, respectively. GMQE is expressed as a number between 0 and 1, and values greater than 0.7 are considered better. QMEAN scores around zero indicate models with high quality. The final 3D structure model of Leptospiral GroEL was similar to that of *E. coli* and *T. thermophilus* with three the domains (equatorial, intermediate, and apical) [32, 33], showing its structural conservation (Fig. 1b).

Multiple sequence alignments were performed by Clustal Omega program, comparing *L.interrogans* GroEL with representative sequences of chaperonin subunit group I, which are found in bacteria and eukaryotic organelles (Fig.S1a) [36]. These analyses reveled a remarkable amino acid sequence conservation between *L. interrogans* GroEL and sequences from *E. coli* (taxid:562), *T. thermophilus* (taxid:274), and human mitochondria (taxid:9606), with 62–64%, 66–68%, and 40–51% of identity, respectively*.* As expected, the Clustal alignments among *L.interrogans* GroEL with representative sequences of chaperonin subunit group II (present in archaea and the cytoplasm of eukaryotes) have shown more sequence divergence mainly in apical domain, which contain the substrate-binding sites (Fig.S1b). The identities of *L.interrogans* GroEL with sequence from *E. coli* (EFS2935563.1), *T. thermophilus* (WP_011174077.1), human TCP1 (NP_110379.2), and Drosophila TCP1 (AAA28927.1) were 64, 68, 22 and 23%, respectively. The TCP-1 ring complex (TRiC; also named CTT) is a large complex composed of two rings of seven to nine proteins similar to TCP1 (T-complex protein 1) [36].

Next, we evaluated the amino acid sequence identity of the *L.interrogans* GroEL with proteins from other leptospires species in NCBI databases by BLASTp program. The results showed that GroEL is highly conserved in strains of *Leptospira* spp., with identity values ranging from 81.8 to 100% (Table S1, Fig. S2).

### Purification of recombinant proteins

The His-tagged recombinant proteins were purified by metal affinity chromatography, and homogeneous bands were visualized by staining the SDS-PAGE with Coomassie Brilliant (Fig.S3a). The truncated form of leptospiral immunoglobulin-like protein B (LigBC, aa 631–115) [9–12] and the leptospiral protein 25 (Lp25) [18, 37] were used as positive and negative controls in binding assays, respectively. LipL31 and the truncated form of leptospiral immunoglobulin-like protein A (LigAC, aa 631–1156) were applied as positive control of cytoplasmatic and outer membrane proteins, respectively [18, 37].

### GroEL is a surface protein and secreted into the extracellular environment

**Fig. 2:**
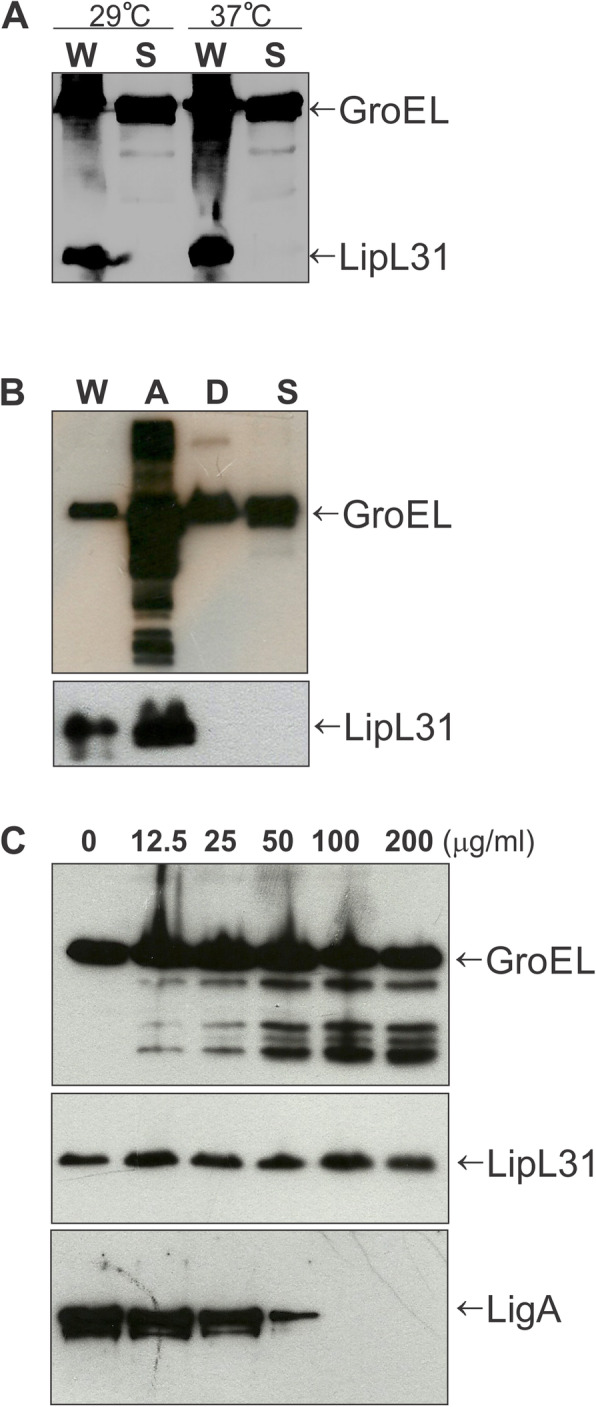
Subcellular localization of the GroEL protein. *L. interrogans* serovar Copenhageni strain Fiocruz L1–130 was cultivated at 29 °C and in conditions that mimics the host environment, with shift temperatures from 29 to 37 °C for 5 h and in osmolarity of 300 mOsm. **a** Whole-cell lysates (W) and cell culture supernatant fractions (S) were analyzed by immunoblotting using anti-GroEL and anti-LipL31 (positive control of cytoplasmatic protein) antisera. **b** Whole cell (W), Triton X-114 fractions (A and D), and culture supernatant fraction (S) were analyzed by immunoblotting with anti-GroEL and anti-LipL31. **c** Proteinase K accessibility assay. Intact leptospires were incubated with different concentrations of proteinase K and processed for immunoblot analyses using antibodies against GroEL, LipL31 or LigA (positive control of outer membrane protein). Full-length blots are shown in the Supplementary Material as Fig. S4

Previous works had demonstrated that GroEL proteins are localized primarily in cytoplasm [21]. However, some studies indicate that, in bacteria, they can be associated with cell membrane, as surface proteins, or secreted into the extracellular environment [22, 26, 31]. To investigate the cellular localization of leptospiral GroEL protein, we first performed immunoblot assays using antisera against GroEL protein and against a previously identified cytoplasmic protein named LipL31 [37, 38] used as a control. Whole-cell and culture supernatant samples were obtained from leptospires maintained at 29 °C and also in conditions that mimics the host environment, with shift temperatures from 29 to 37 °C for 5 h and in osmolarity of 300 mOsm [39, 40]. GroEL was detected in whole cell extract and culture supernatant fraction, indicating its presence as a secreted protein at 29 °C and 37 °C. As expected, LipL31 was found only associated to whole-cell (Fig. 2a). In addition, we assessed the GroEL cellular localization using Triton X-114 detergent fractionation of leptospires that were cultured in the temperature shift and 300 mOsm. GroEL protein was detected in all fractions: in the whole-cell extract (W), in the aqueous phase (A) that contains mainly periplasmic proteins, in detergent phase (D) which consists of proteins associated with outer membranes, and in the culture supernatant (S). Whereas LipL31 was observed in the whole-cell (W) and aqueous phase (A), again it was not detected in the detergent fraction (D) or in the supernatant (S) (Fig. 2b).

Lastly, we also investigated the surface localization of GroEL protein using the proteinase K proteolysis of intact leptospires. Figure 2c shows that GroEL was susceptible to protease treatment in a dose-dependent manner, suggesting that this protein is exposed on the surface of bacteria. The cytoplasmatic LipL31 protein was not affected, while leptospiral immunoglobulin-like (Lig) protein A (LigA), a previously characterized outer membrane protein, was completely degraded with concentration greater than 50 μg/mL of proteinase K in our assay conditions. The susceptibility of GroEL, LigAC and LipL31 recombinant proteins to proteinase K treatment was tested, as shown in the Fig. S3b. These results demonstrated that the proteinase K assay functioned adequately, and also suggested that a fraction of GroEL is localized and exposed on the leptospire surface. Full-length blots of Fig. 2 are shown in the Supplementary Material as Fig. S4.

### GroEL binds extracellular matrix and plasma proteins

**Fig. 3:**
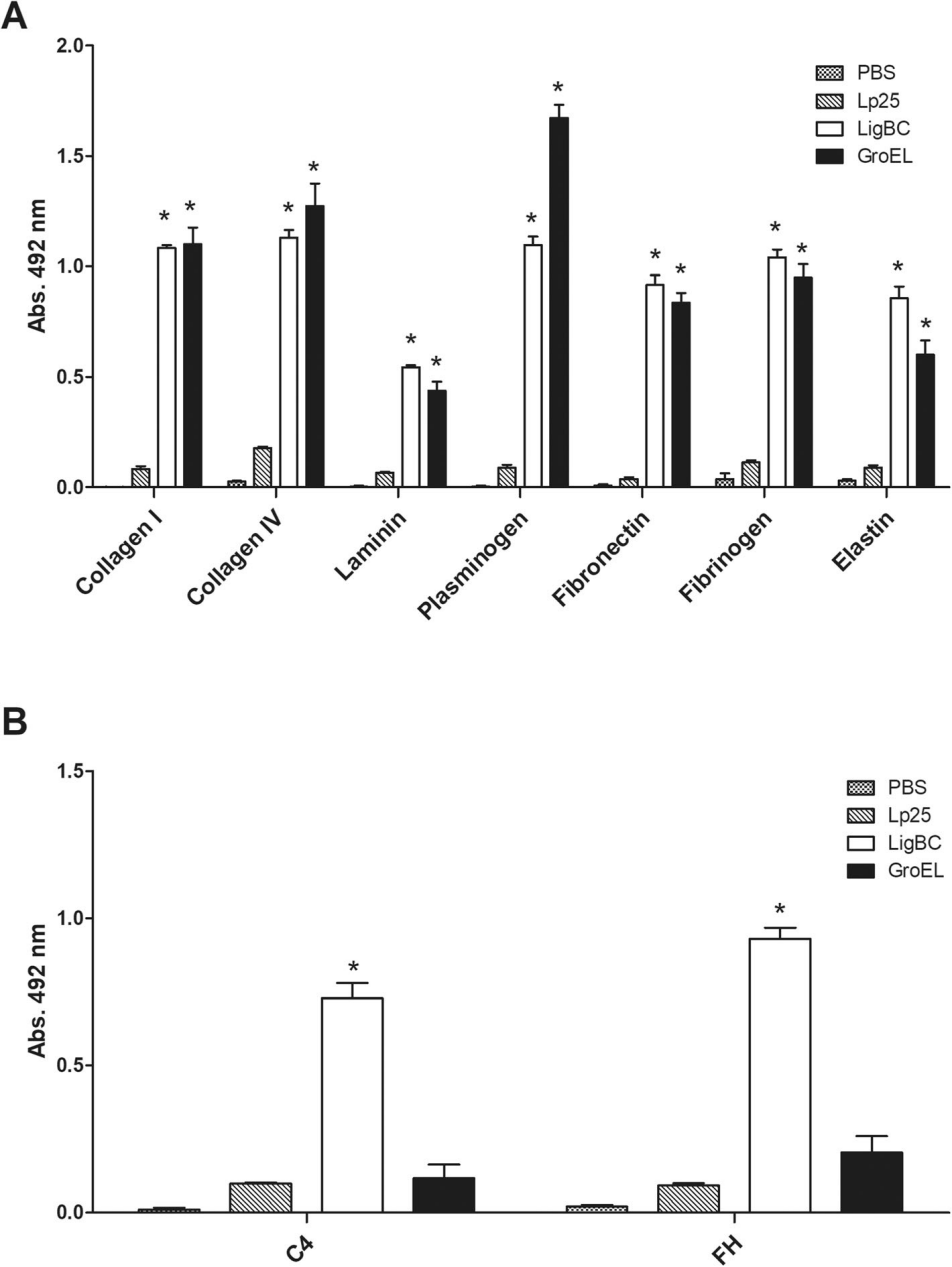
Binding of GroEL to the host components. Protein adherence to collagen type I, collagen type IV, laminin, plasminogen, plasma fibronectin, fibrinogen, elastin **(a)**, C4 and FH **(b)** was assessed by an ELISA-based assay. One micromolar of recombinant protein was added to wells previously coated with the target proteins. Lp25 and LigBC were used as negative and positive binding controls, respectively. The absorbance values obtained with BSA were subtracted from values obtained with binding of the recombinant proteins to host components. Data represent the mean of absorbance value at 492 nm ± standard error of three independent experiments. For statistical analyses, the binding of GroEL protein to host components was compared to the binding of the negative-control protein Lp25 by *t-*test. Values of *p* < 0.05 (*) were considered statistically significant

**Fig. 4:**
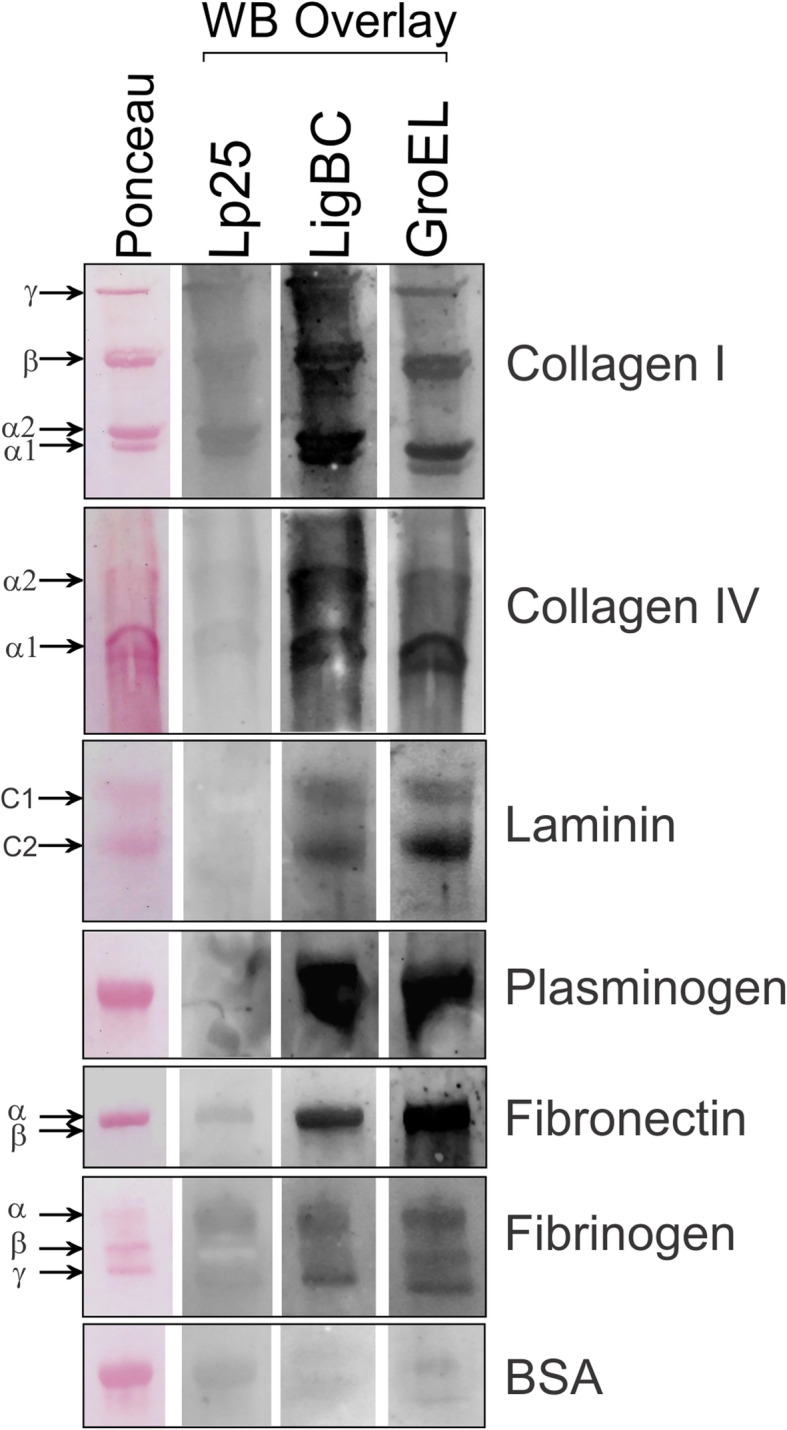
Western Blot Overlay. Collagen type I, collagen type IV, laminin, plasminogen, plasma fibronectin, fibrinogen, and BSA (negative control) were subjected to 4–20% gradient gel (SDS PAGE) under reducing conditions, transferred to nitrocellulose membranes and stained with Ponceau S (right). After incubation of membrane with recombinant proteins, the binding was detected with polyclonal antibodies recognizing Lp25, LigBC and GroEL. Chains of host proteins are indicated by γ (400 kDa), β (270 kDa), α2 (130 kDa), α1 (115 kDa) of collagen type I; α1 (140 kDa), α2 (160 kDa) of collagen type IV; C1 (400 kDa) and C2 (200 kDa) of laminin; α (220 kDa) and β (212 kDa) of plasma fibronectin; α (63.5 kDa), β (56 kDa), γ (47 kDa) of fibrinogen; plasminogen and BSA with 70 kDa and 66 kDa, respectively. Lp25 and LigBC were used as negative and positive binding controls, respectively. Full-length Western blot overlays are shown in the Supplementary Material as Fig. S5

To evaluate a putative capacity of the GroEL protein to interact with host proteins components, different targets (collagens I and IV, laminin, elastin, plasma fibronectin, plasminogen, fibrinogen, C4 and FH) were immobilized onto microplate wells and the binding was analyzed by enzyme-linked immunosorbent assay (ELISA) after incubation with GroEL protein. As shown in the Fig. 3, GroEL exhibited significant levels of binding activities (*p* < 0.05) to collagens I and IV, laminin, elastin, plasma fibronectin, plasminogen, fibrinogen (Fig. 3a). These results are comparable with those obtained using LigBC protein as positive control [9–12]. GroEL did not interact with C4 and FH (Fig. 3b). In contrast, Lp25 protein [8, 37], used as negative control, did not exhibit significant levels of binding activities to these host ligands. We have previously shown that Lp25 did not bind laminin, collagens I and IV, fibronectin, C4, and FH [8]. In the current study, we found that Lp25 protein also did not interact with elastin, plasminogen, and fibrinogen (Fig. 3a). Western blot overlay (WBO) assays were performed to demonstrate the binding specificity of GroEL to host proteins. Host proteins were subjected to SDS-PAGE, transferred to nitrocellulose membranes, and examined for the ability to bind to soluble recombinant proteins. As shown in Fig. 4, GroEL was able to attach to collagens I and IV, laminin, plasminogen, plasma fibronectin, and fibrinogen. These results are comparable with those obtained by Elisa and with LigBC protein as positive control in the WBO assays. Negligible or no binding to host proteins was detected with Lp25, used as non-adhesin leptospiral protein or when BSA, used as host protein, was included as negative controls in the WBO assays. Full-length blots of Fig. 4 are shown in the Supplementary Material as Fig. S5.

**Fig. 5:**
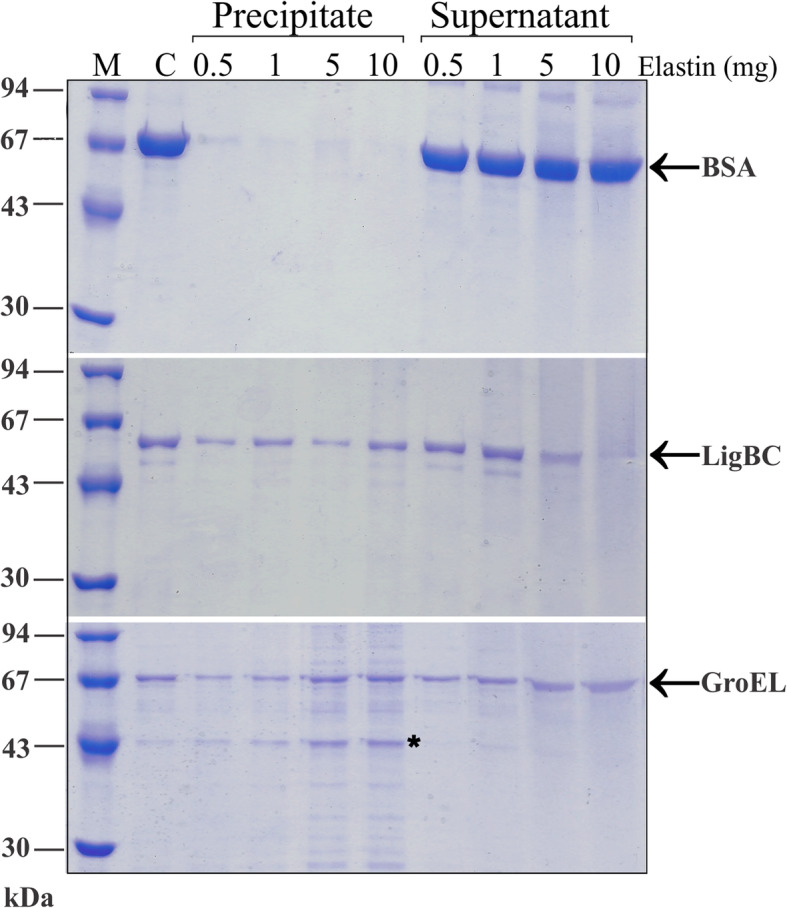
Binding of recombinant proteins to elastin. The ability of recombinant proteins to bind to insoluble elastin was evaluated by SDS-PAGE. BSA was used as a negative control. The bound (precipitate) and unbound (supernatant) fractions were analyzed by 12% SDS-PAGE. M: molecular mass protein markers. C: control protein. The asterisk (*) correspond to a product of GroEL degradation that was completely absorbed to the elastin

The elastin is an insoluble protein and the elastin-binding ability of GroEL to bind it was assessed by SDS-PAGE after incubation to elastin. The fractions of bound (precipitate) or unbound proteins (supernatant) to the insoluble elastin were obtained by centrifugation and analysed by SDS-PAGE. As shown in Fig. 5, the amount of LigBC and GroEL proteins in the precipitate fraction increased, while the amount of unbound protein in the supernatant decreased, confirming that these proteins can bind to elastin. It was also possible to observe the presence of a band with ~ 43 kDa that can correspond to a degradation product of GroEL. Interestingly, this ~ 43 kDa band was completely absorbed to the elastin. In contrast, the BSA did not bind to the elastin, since none was found in the precipitate fraction. In LigBC, it was clear that higher amount of elastin was required to better observe their interaction. At 5–10 mg of elastin, it was observed a higher amount of LigBN in the precipitate (bound) than in the supernatant (unbound). In contrast, BSA, used as negative control, did not bind to elastin, being equally presented in only the supernatant fraction.

**Fig. 6:**
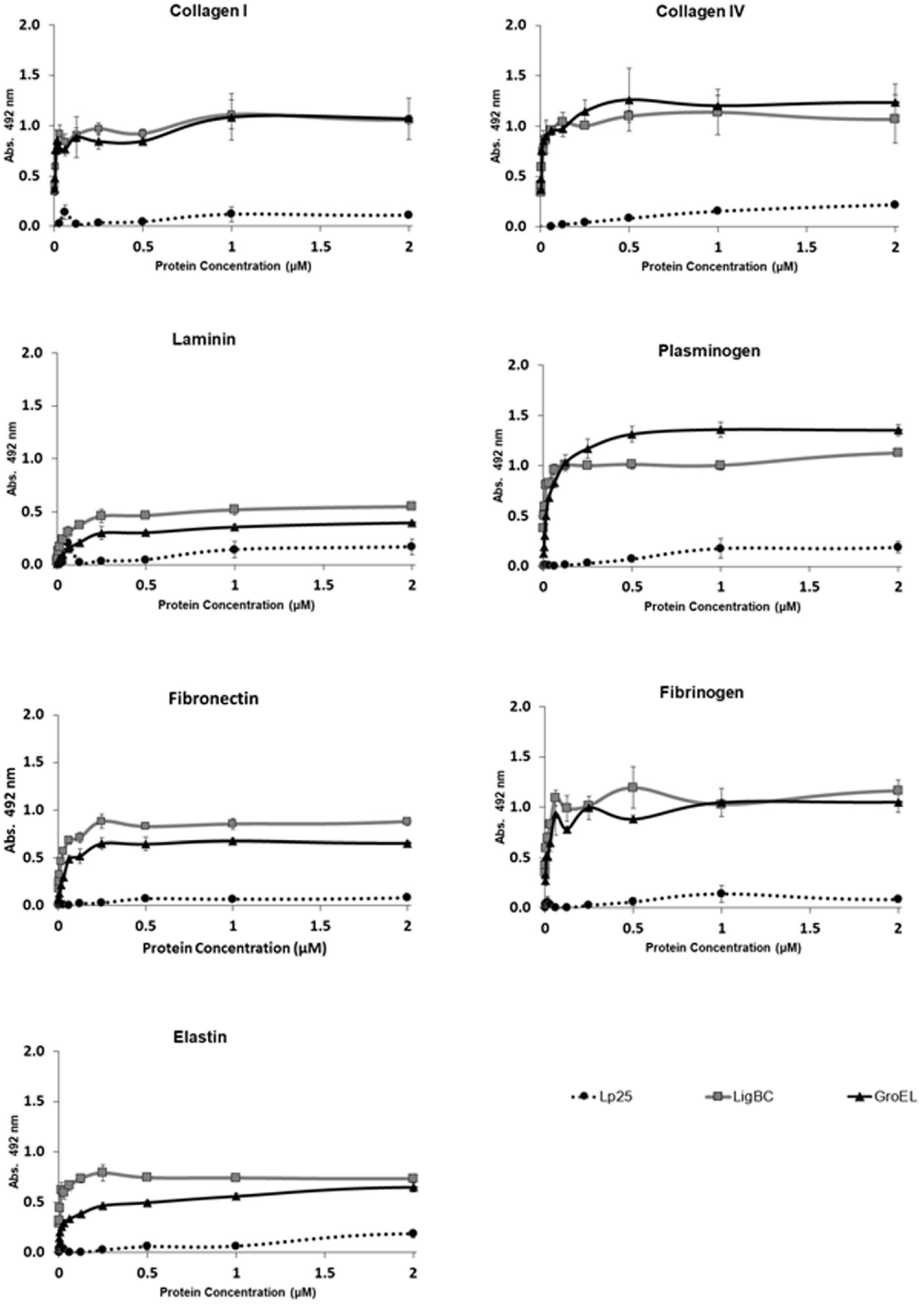
Binding of GroEL to host molecules as a function of protein concentration. The dose-dependent binding was determined by varying the protein concentration from 0 to 2 μM. The absorbance values obtained with BSA were subtracted from values obtained with the binding of recombinant proteins to host components. Each point represents the mean absorbance value at 492 nm ± standard deviation of three independent experiments. Lp25 and LigBC were used as negative and positive binding controls, respectively

In order to further characterize the interaction between GroEL and host proteins, quantitative assays were performed as shown in the Fig. 6 and Table 1. A dose-dependent binding to all tested ligands was observed with an increasing concentration of GroEL (0–2 μM) (Fig. 6). These results showed that this protein binds to tested host proteins in a concentration-dependent manner. The low K_D_ values obtained for GroEL protein indicated a high affinity with these host components (Table 1). For comparison, the results obtained from LigBC (positive control) and Lp25 (negative control) proteins were also presented. As expected, Lp25 displayed no binding activity with host components. The K_D_ values for GroEL were similar to those obtained with LigBC protein.

**Table 1:** Dissociation constants (μM) of GroEL binding to host molecules

	GroEL	LigBC (positive control)	Lp25 (negative control)
**Collagen I**	0.011 ± 0.004	0.008 ± 0.002	0.699 ± 0.587
**Collagen IV**	0.006 ± 0.001	0.007 ± 0.001	2.420 ± 0.001
**Laminin**	0.115 ± 0.012	0.041 ± 0.003	1.136 ± 0.959
**Plasminogen**	0.031 ± 0.002	0.005 ± 0.001	4.433 ± 0.074
**Plasma**	0.034 ± 0.003	0.015 ± 0.002	5.368 ± 0.959
**Fibronectin**	0.010 ± 0.001	0.006 ± 0.001	6.3690 ± 3.608
**Fibrinogen**	0.023 ± 0.003	0.004 ± 0.001	0.606 ± 8.044
**Elastin**	0.023 ± 0.003	0.004 ± 0.001	0.606 ± 8.044

**Fig. 7:**
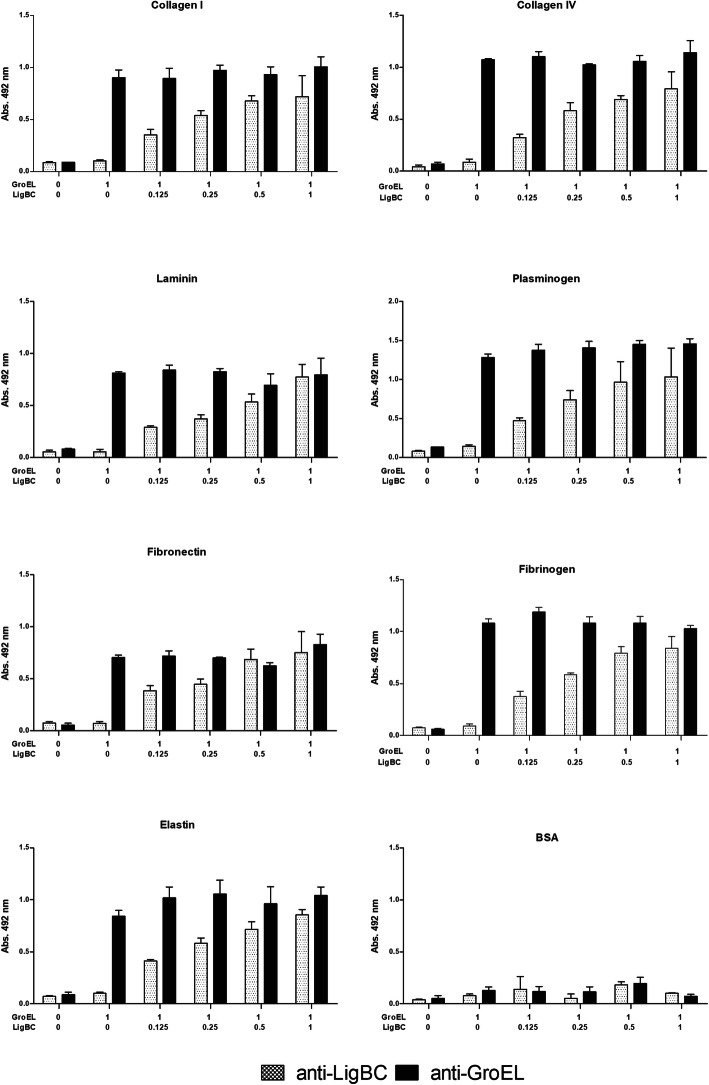
Competitive binding essays. Different amounts of LigBC (0–1 μM) and a fixed amount of GroEL (1 μM) were added to immobilized host proteins. Concentrations are indicated below the graphs. Bound GroEL and LigBC were detected using mouse anti-GroEL (black boxes) and anti-LigBC (gray boxes), respectively, followed by peroxidase-conjugated secondary antibodies. Optical densities were determined at 492 nm. Each point represents the mean absorbance value at 492 nm ± standard deviation of three independent experiments

Next, we investigated whether GroEL and LigBC would compete for the same binding sites on host proteins. Collagens I and IV, laminin, elastin, plasma fibronectin, plasminogen, fibrinogen were immobilized in microtiter wells and a competitive ELISA were performed by adding a fixed amount of GroEL (1 μM) and different quantities of LigBC (0-1 μM). In the Fig. 7, it is possible to observe that the binding of GroEL or LigBC to host proteins was not altered in the molar ratios evaluated. Another competitive binding assay was performed fixing the amount of LigBC protein and varying the GroEL content (data not shown), and we also observed no competition for binding to proteins. These results indicate that these proteins have distinct binding sites on these host proteins.

### GroEL binds to Vero cells

**Fig. 8:**
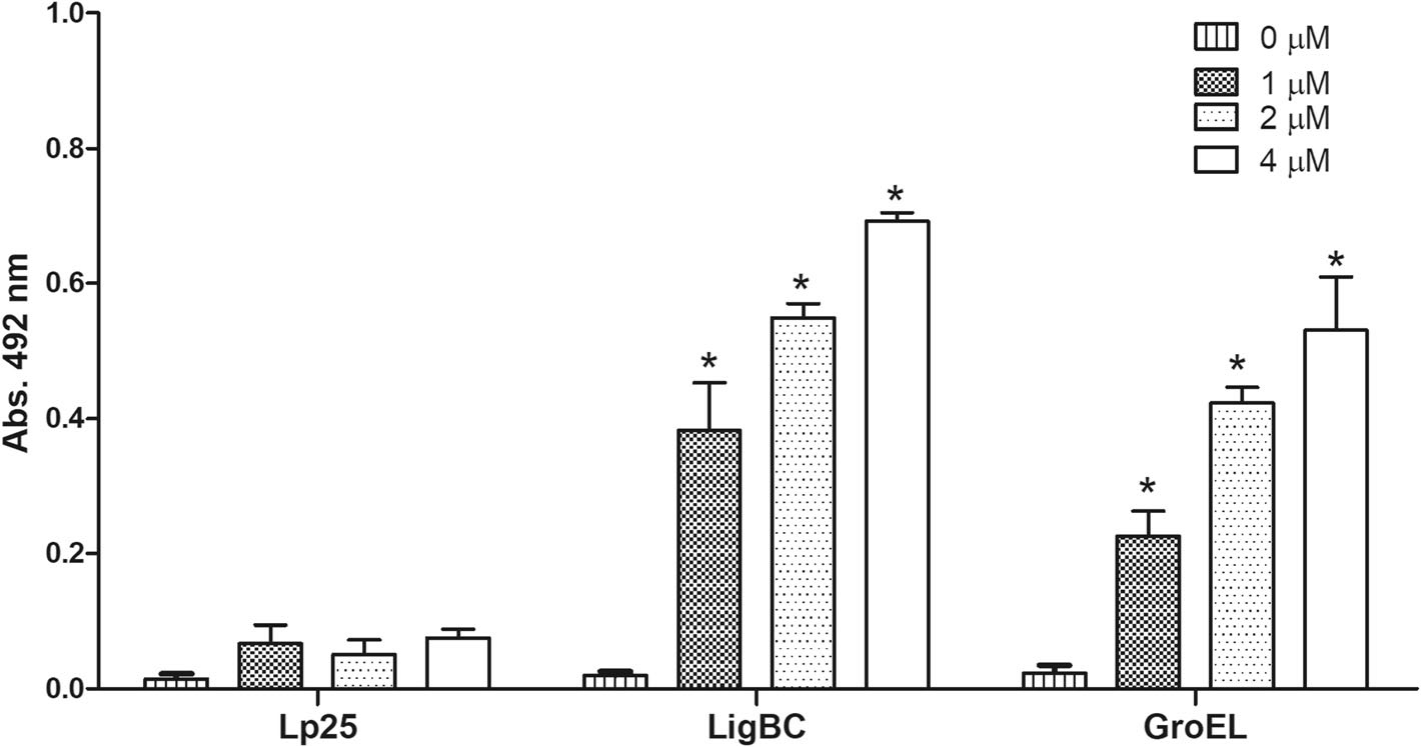
Binding of GroEL to the Vero Cells. Protein adherence to cells was assessed by an ELISA-based assay. Zero to four micrometres recombinant proteins were added to wells previously coated with cells. Lp25 and LigBC were used as negative and positive controls, respectively. Data represent the mean of absorbance value at 492 nm ± standard error of three independent experiments. For statistical analyses, the binding of GroEL protein to the Vero cells was compared to the binding of the negative-control protein Lp25 by t-test. Values of *p* < 0.05 (*) were considered statistically significant

We have also tested GroEL protein for the ability to bind to Vero cell cultures. As shown in Fig. 8, GroEL was able to significantly bind to cells. Lp25 demonstrated no significant binding to these cells, while LigBC had significantly higher binding activity as compared to negative control (medium). These results are in accordance with those obtained using LigBC protein as positive control [41].

### GroEL induces TNF-α and IL-6 in murine macrophage J774.1 cells

**Fig. 9:**
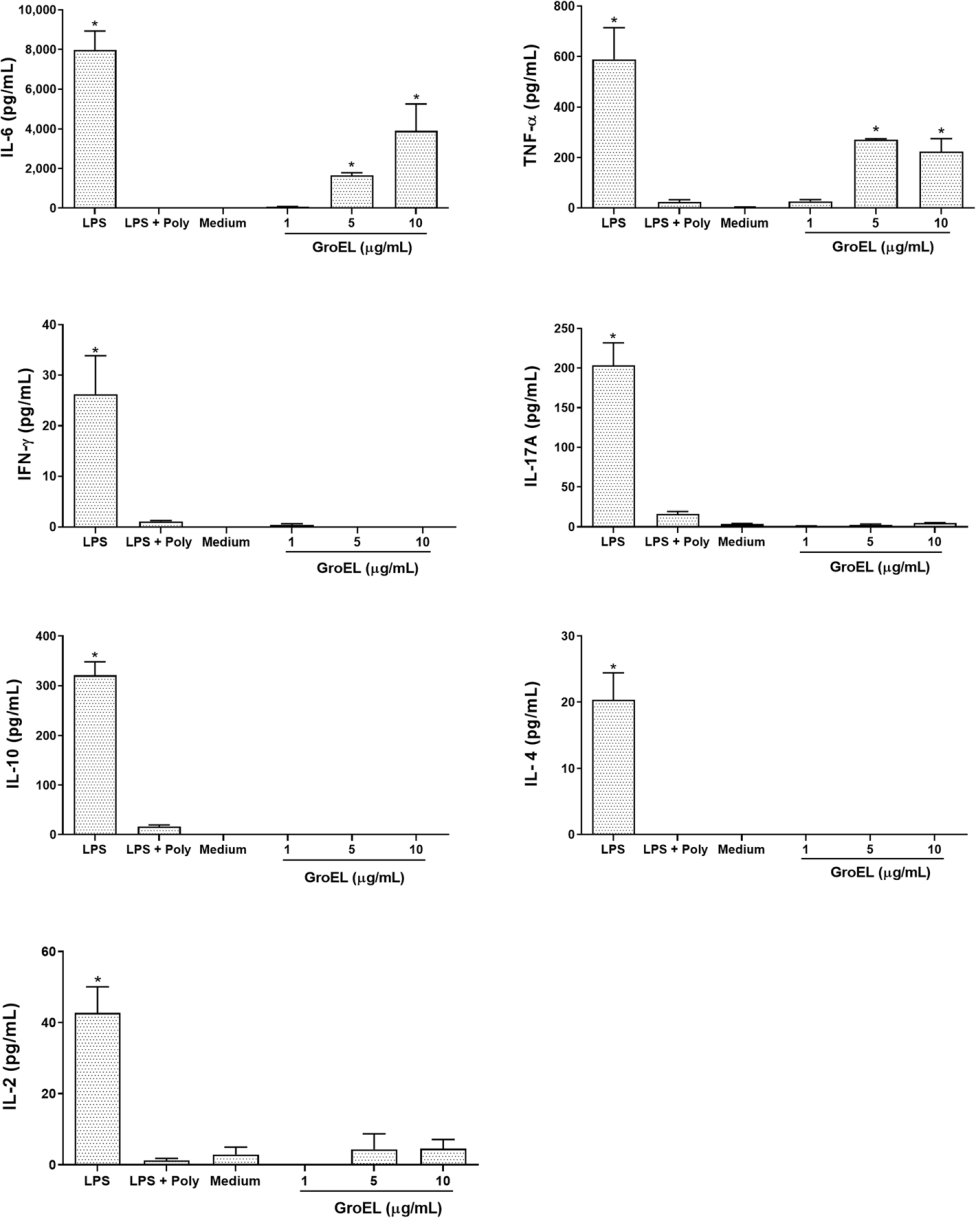
Cytokine secretion assay. J774A.1 cells were cultured in the presence of different amounts of GroEL protein and 50 ng/mL LPS treated and untreated with 50 μg/mL of polymyxin B. The concentrations of interleukin-10 (IL-10), interleukin-17A (IL-17A), tumor necrosis factor (TNF-α), interferon-γ (IFN-γ), interleukin-6 (IL-6), interleukin-4 (IL-4) and interleukin-2 (IL-2), in culture media, were measured by flow cytometry. Cells were stimulated with 0 (medium), 1, 5 and 10 μg/mL of GroEL after 20 h of incubation at 37 °C. Data represent the mean of cytokine concentrations in the cellular supernatant ± standard error of three independent experiments. Values of *p* < 0.05 (*) were considered statistically significant, when compared to the binding of the cells did not stimulate (medium) by t-test

**Fig. 10:**
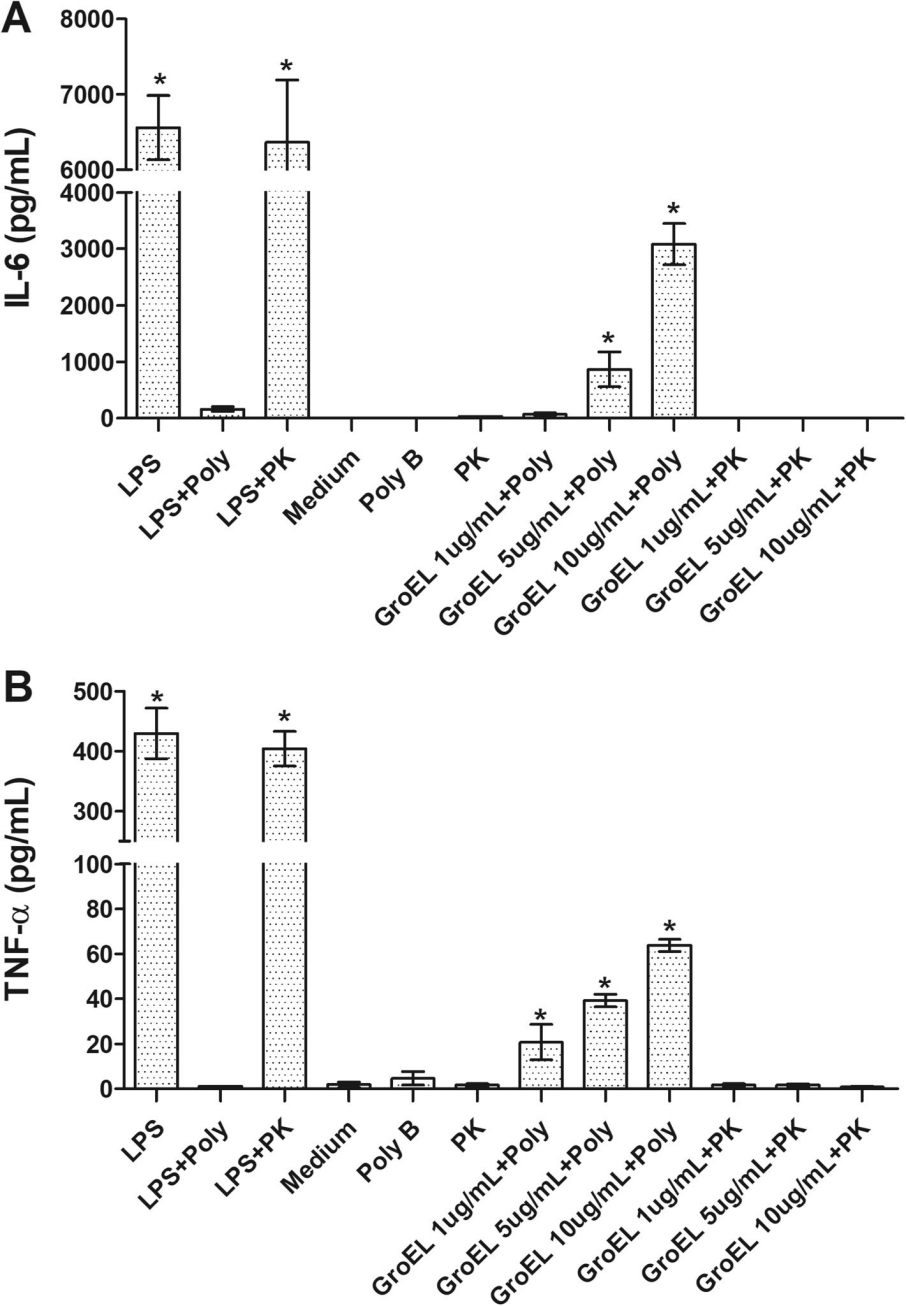
Cytokine secretion assay. J774A.1 cells were stimulated (20 h of incubation at 37 °C) with GroEL (10 μg/mL) and LPS (50 ng/mL) treated with 50 μg/mL of polymyxin B or 50 μg/mL of proteinase K. Data represent the mean of IL-6 **(a)** and TNF-α. **(b)** concentrations in the cellular supernatant ± standard error of three independent experiments. Values of *p* < 0.05 (*) were considered statistically significant, when compared to the binding of the cells did not stimulate (medium) by t-test

Since several bacterial HSPs have been identified as potent proinflammatory agents [24–28], we investigated the cytokine expression profiles of murine macrophage cell line J774.1 stimulated with different concentrations of GroEL protein. As shown in the Fig. 9, of the seven cytokines simultaneously measured, only TNF-α and IL-6 were induced by GroEL. The expression of TNF-α and IL-6 was significantly higher when compared to unstimulated cells (*p* < 0.05). The stimulation of these two cytokines were specific in a dose-dependent manner, Fig. 10 shows significant stimulation of TNF-α and IL-6 after 20 h in contact with increasing amounts of GroEL protein (0–10 μg/mL). The specificity of these results was confirmed with pre-treatment of GroEL with polymyxin B or proteinase K. The activation of J774.1 cells with GroEL treated with polymix B induced the production of TNF-α and IL-6, in a dose dependent manner. In contrast, the treatment of LPS with polymyxin B reduced these cytokines to the level of unstimulated cells, indicating that the induction of TNF-α and IL-6 was specific. Moreover, the treatment of GroEL protein with proteinase K inhibited the TNF-α and IL-6 productions. As expected, the treatment of LPS with proteinase K did not inhibit the production of these cytokines. These results confirmed that GroEL induces TNF-α and IL-6 in J774.1 cells (Figs. 9 and 10).

## Discussion

Leptospiral infection begins by the host penetration through mucous membranes or broken skin that is in contact with contaminated water or soil [1, 2]. Once in the bloodstream, leptospires are able to escape from immune responses, disseminate and colonize target organs, mainly kidneys and liver, causing the disease [2]. However, the pathogenic mechanisms of leptospirosis are still not completely understood.

In this work, the capacity of leptospiral GroEL protein to bind in vitro to Vero cells and to collagens I and IV, laminin, elastin, plasma fibronectin, plasminogen, and fibrinogen in a dose-dependent and saturable manner, was described. Moreover, the production of IL-6 and TNF- α in J774.1 murine macrophage cell line stimulated by this protein was also observed. These results implicate a potential role of leptospiral GroEL in the host-leptospire interactions and pathogenesis.

Pathogen adherence to host cells and tissues is the first and essential event in the establishment of the infection, followed by evasion of immune defense, invasion, dissemination and persistence [41–43]. Pathogenic bacteria present a large number of adhesins on their surface that mediate specific interactions with host cell receptors and/ ECM components [43]. ECM is a biologically active network composed of a complex mixture of macromolecules able to establish interactions between the different components and cells. This organization is formed by collagens, laminin, elastin, fibronectin, vitronectin, proteoglycans, glycosaminoglycans, and other glycoproteins, which interact three-dimensionally and perform important cellular functions, such as morphogenesis, signaling, repair and migration [44]. Most pathogenic bacteria utilize the EMC molecules as targets for adhesion and invasion processes. Some of them are capable of degrading ECM proteins, using their proteases or host proteases, as plasminogen/plasmin and matrix metalloproteinases, leading to inflammatory responses and tissue damage [45–47]. Plasminogen is a proenzyme present in the plasma that is converted to plasmin by tissue-type plasminogen activator or urokinase (uPA). Although plasmin is considered the main protease of the fibrinolytic system, it also cleaves other substrates, such as ECM proteins and fibrin clots [48]. Several pathogens present different strategies of interacting with plasminogen, including plasminogen-binding proteins that can promote the conversion to plasmin, in the presence of uPA. The degradation of EMC proteins and cell-junction proteins facilitate the bacterial invasion and colonization processes [48, 49]. Damage in some of these molecules can implicate in severe consequences for the host health. For example, fibrinogen is a glycoprotein found in high concentrations in the plasma, which acts in the coagulation cascade and in the wound healing process. It is cleaved by thrombin and converted into fibrin, forming the clot. Adherence of bacteria to fibrinogen could interfere with the hemostasis and the healing process, facilitating the pathogen dissemination [49, 50].

A large number of proteins from *Leptospira* spp. that interact with ECM components have been identified to date [7–13]. For instance, leptospiral endostatin-like protein (Lsa24, also named as LenA /LfhH) is able to bind to laminin [8, 51], LipL32 adheres to laminin, fibronectin and collagen IV [52, 53]. Leptospiral binding proteins for plasminogen have also been reported [15, 18, 54, 55]. LemA [15], LipL32 [52], elongation factor Tu (EFtu) [54], and enolase [55] are also able to attach to plasminogen which facilitate it conversion to plasmin. Some proteins that bind to fibrinogen have been identified, including OmpL1 [50]. LigA and LigB are multifunctional proteins capable of interacting with various EMC and plasma proteins [9–12, 18, 56, 57]. Here, GroEL from *Leptospira* spp. was able to adhere to host proteins with high avidity, comparable to LigBC protein used as a positive control (Table 1). LigBC corresponds to half of immunoglobulin-like repeat 7 plus domains 8 to 12 [18, 56]. It has been previously reported that other constructions containing the same LigB-specific immunoglobulin-like repeats, named LigB U1 (aas 631–1125) and LigBCen (aas 631–1417), bind to fibrinogen, plasminogen and several EMC proteins, including collagens I and IV, laminin, elastin and fibronectin [9–12, 18, 56, 57].

In addition, we have demonstrated that GroEL is a surface protein, and it is secreted extracellularly. Previous reports have shown that several members of bacterial HSPs are also secreted proteins and able to interact with Vero cell monolayers and cell components, ECM, plasminogen and other host molecules [23, 58–62]. GroEL from *Mycoplasma pneumonie* is a surface protein, which adheres to plasminogen, vitronectin, fibronectin, fibrinogen, lactoferrin and laminin [59]. In enteropathogenic *Escherichia coli*, GroEL is secreted and mediates in vitro binding to cellular fibronectin [60]. A 66-kDA heat shock protein is involved in the adherence of *Salmonella typhimurium* to intestinal mucus [61]. HSP70 was found on the cell surface of *Mycobacterium tuberculosis* and it is also a plasminogen binding protein [62]. The surface localization as well as its presence secreted in the supernatant contribute to the hypothesis that GroEL from *Leptospira* may have important functions in the infectious process.

Supporting this idea, our results from cytokine secretion assay revealed that GroEL induced the synthesis of proinflammatory cytokines IL-6 and TNF-α in cultured macrophages. These data are consistent with other studies. Volz et al. [63] demonstrated significant production of TNF-α by patient’s CD4^+^T cells stimulated with leptospiral antigens. The levels of TNF-α were higher in CD4^+^T cells obtained from patients with severe and life-threatening manifestations of leptospirosis than in those from asymptomatic and heathy control subjects. Synthesis of TNF-α was also noted in human peripheral blood mononuclear cells cultured in the presence of leptospire antigens [64]. Other protein from *Leptospira* spp. are known to induce the production of cytokines [64–67]. Wang et al. [67] showed that leptospiral hemolysins induce strong production of IL-1β, IL-6 and TNF-α in human and mouse macrophages. Some members of the microbial HSPs family are also potent inducers of pro-inflammatory cytokines, such as HSP60, HSP65, HSP70, HSP90 [25–29]. GroEL from *Escherichia coli* induces IL1β and Il-6 in human monocytes [24]; and exposure of monocytes to *M. leprae* hsp65 stimulates production of TNF-α, IL-6 and IL-8 are some other examples [68].

Interestingly, IL-6, and TNF-α cytokines have been considered markers for the severity of leptospirosis, since they are found at significantly higher levels in plasma samples from fatal cases and from patients with severe manifestation of this disease compared with samples from mild cases and heathy subjects [69]. TNF-α is a proinflammatory cytokine produced in response to infections and is considered a primary mediator of the innate immune system. However, when its production is excessive and prolonged, TNF-α becomes harmful to the organism, causing dysregulation of the immune response, inducing activation of other cytokines, as well as the cellular oxidative system, which promotes potentially lethal inflammation and tissue damage [69]. The production of IL-6 occurs shortly after that of TNF-α, already at the beginning of the inflammatory process. It is one of the principal mediators of the acute inflammatory phase. Based on these data, it has been proposed that pathophysiology of leptospirosis might be associated with elevated release of cytokines and proinflammatory citokines during infection [69, 70]. This “cytokine storm” causes persistent inflammation, tissue injury and organ failures, as observed in sepsis [70, 71]. The secretion of GroEL is in accordance with the observation of its role in the production of IL-6 and TNF-α as described here and may associate the level of GroEL to leptospirosis severity due to the level of these cytokines and represent a potential clinical relevant marker of the disease as well as a potential lead target for leptospirosis therapy.

The multifunctional HSPs belong to an abundant group of proteins named as moonlightings, which was primarily described as intracellular proteins. They participate in basic cellular functions, but also have other unrelated roles generally associated with pathogenicity when localized on the cell surface or into the extracellular environment [71, 72]. It should be noted that none of these proteins possesses any known signal sequences or motifs for secretion and the mechanisms by which GroEL and other moonlighting proteins are secreted are still not established [23, 71, 72].

Moreover, sequence comparison of *L.interrogans* GroEL with representative chaperonin subunits from group I (present in bacteria and endosymbiotic organelles) revealed remarkable amino acid conservation. Unlike, most sequence divergence was observed in the alignments of *L.interrogans* GroEL to sequences from group II chaperonin subunits (found in Archaea and eukaryotic cytosol), mainly in the substrate-binding domain. Group I and II chaperonin subunits present the same basic structure composted by apical, intermediate and equatorial domains. Despite this structural conservation, previous reports have shown additional functions of chaperonins besides those to assist protein folding and to protect proteins from denaturation [21–31]. The divergent sequences observed in the substrate-binding domains of chaperonins from group I in comparison of sequences from group II chaperonin subunits might be related with the multiple unexpected biological activities of bacterial HSPs that have been identified, such as induction of proinflammatory molecules [24–28] and adheshion of the host components [23, 58–62], as described here. In conclusion, leptospiral GroEL protein may contribute to the adhesion of leptospires to host tissues, stimulate the production of cytokines during infection and be involved in the pathogenesis of leptospirosis.

## Conclusions

Taken together, our data show that GroEL of *Leptospira spp*. is a moonlighting protein, including the new adhesin activity with multi-binding properties to host proteins and to target cells. It also has the capacity to stimulate proinflammatory cytokine productions that might contribute to the pathogenesis and severity of leptospirosis. Moreover, it is conceivable that the different moonlighting activities of leptospiral GroEL is related to its localization on the cell surface or secreted extracellularly. These features might indicate an important role of GroEL in the pathogen-host interaction in the leptospirosis.

## Methods

### Reagents and antibodies

Minimum Essential Medium (MEM, Gibco®), Dulbecco’s modified Eagle’s medium (DMEM, Gibco®), L-glutamine, trypsin, fetal bovine serum (FBS, Gibco®), ortho-phenylenediamine dihydrochloride, isopropyl-1-thio-β-D-galactopyranoside (IPTG), Ellinghausen-McCullough-Johnson-Harris (EMJH) medium, Tween-20, penicillin, and streptomycin were purchased from Thermo Fisher Scientific, (Boston, MA, USA). Bovine serum albumin (BSA), lipopolysaccharides (LPS) from *E. coli* 026/B6, Triton X-114, anti-mouse IgG antibody labelled with horseradish peroxidase, proteinase K, polymyxin B sulfate salt, collagen Type I (purified from rat tail), collagen Type IV and laminin (purified from basement membrane of Engelbreth-Holm-Swarm mouse sarcoma), elastin (purified from bovine neck ligament), fibronectin, plasminogen and fibrinogen (purified from human plasm) were products of Sigma-Aldrich (St. Louis, MO, USA). Human FH and C4 were purchased from Complement Technology (Texas, USA).

### Bioinformatic analysis

The Conserved Domain Database (CDD) web server (https://www.ncbi.nlm.nih.gov/Structure/cdd/wrpsb.cgi) was used to identify putative conserved domains in GroEL sequences. Tertiary structure prediction was performed by SWISS-MODEL protein homology modeling server (https://swissmodel.expasy.org/). Multiple sequence alignments were performed by Clustal Omega program (https://www.ebi.ac.uk/Tools/msa/clustalo/). The accession numbers of representative sequences of chaperonins are AAS69936.1 (*L.interrogans*), EFS2935563.1 (*E.coli*), WP_011174077.1 (*T. thermophilus*), NP_110379.2 (human TCP1), AAA28927.1 (*Drosophila* TCP1) found in https://www.ncbi.nlm.nih.gov/. The data generated by bioinformatic analysis and the sequences used in the multiple alignments are available in the Butantan Institute Repository [https://repositorio.butantan.gov.br/handle/butantan/3635].

### Cloning, expression and purification of recombinant proteins

The coding sequence of the GroEL protein (AAS69936.1, 60 kDa) was amplified by PCR from genomic DNA of *L. interrogans* serovar Copenhageni strain Fiocruz L1–130 using the primers F: GGATCCGCGAAAGATATTG and R: AAGCTTTTACATCATTCCG. After digestion with *Bam*HI and *Hind*III restriction enzymes, the fragment was cloned into the pAE expression vector [73]. The coding sequences of the Lp25 (AAS68646.1; 27 kDa), LipL31 (AAS70054.1, 30 kDa), LigAC (amino acids 631–1225, AAS69086.1; 63 kDa) and LigBC (amino acids 631–1156, AAS69085, 56 kDa) proteins were cloned into the pAE vector as previously described [37, 39, 73, 74]. The protein expression was induced with 1 mM IPTG at 37 °C for 3 h in *E.coli* BL21(DE3) strain. The His-tagged recombinant proteins were purified by metal affinity chromatography as previously described [74]. The lipopolysaccharides (LPS) of purified recombinant proteins was removed using the Detoxi-Gel column (Pierce, Rockford, IL) according to the manufacturer’s instructions. The LPS level was determined with the chromogenic *Limulus* amebocyte lysate assay kit (Lonza), and the purified proteins showed approximately 2 EU/μg of protein.

### Production of polyclonal antisera

Polyclonal antibodies against recombinant proteins were generated by immunizing mouse thrice with 10 μg of each purified protein and with aluminum hydroxide as adjuvant. Two weeks after the last immunization, mice were bled from the retro-orbital plexus after isoflurane anesthesia. The anesthetic procedure was performed using a chamber (500 ml volume), containing a moistened cotton pad with 0.5 mL of liquid isoflurane. The soaked pad was placed into the bottom of container separated from animal by a screen, avoiding the direct contact of the animal with isoflurane. At the end of blood collection, each animal was immediately euthanized by prolonged exposure to isoflurane. Animals were monitored for confirmations of death such as cessation of respiration, heart beat and pupillary response to light.

### Binding assay

ELISA was performed to evaluate the binding of GroEL protein to host components. Microtiter plates were coated with 1 μg of collagen I and IV, laminin, plasminogen, fibronectin, fibrinogen, elastin or BSA, overnight at 4 °C, in 0.1 ml phosphate buffered saline (PBS), pH 7.4. The choice of the targets was based on previously published studies that have identified a large number of proteins from *Leptospira* spp. and other bacteria that interacts with collagen I and IV, laminin, plasminogen, fibronectin, fibrinogen, and elastin [7–13, 31, 46–57]. After two washes with PBS-0,05% Tween-20 (PBS-T), nonspecific binding sites were blocked with 1% BSA in PBS, for 2 h at 37 °C. The wells were washed twice with PBS-T and 1 μM of each purified recombinant protein (GroEL, Lp25 or LigBC) were added to wells and incubated for 2 h at 37 °C. Lp25 and LigBC were used as negative and positive binding controls, respectively. After, six washes with PBS-T, the protein binding was detected with mouse anti-recombinant proteins at a 1:5000 dilution followed by peroxidase-conjugated anti-mouse IgG at a 1:5000 dilution. The substrate reaction was performed with ortho-phenylenediamine dihydrochloride and absorbance was measured at 492 nm. The dose-dependent binding was determined by varying the protein concentration from 0 to 2 μM. The absorbance values with BSA were subtracted from values obtained with binding of recombinant proteins to host components. The apparent K_d_ for saturation binding was estimated with the half-maximal binding concentration of recombinant protein, using GraphPad Prism 6 program. For competitive binding essays, collagens I and IV, laminin, elastin, plasma fibronectin, plasminogen, fibrinogen, and BSA (1 μg/well) were immobilized in microtiter wells and a competitive ELISA were performed by adding a fixed amount of GroEL or LigBC (1 μM) and different quantities of LigBC or GroEL (0-1 μM).

### Western blot overlay

Host proteins (collagen type I, collagen type IV, laminin, plasminogen, plasma fibronectin, fibrinogen), BSA, and recombinant proteins were subjected to SDS-PAGE (4–20% Mini-PROTEAN TGX Precast Protein Gel, Bio-Rad) under reducing conditions and transferred to a nitrocellulose membrane. Nonspecific binding sites were blocked by using 10% (w/v) dry milk in PBS-0,05% Tween (PBS-T) at 4 °C. The membrane was rinsed three times with PBS-T for 5 min and was incubated for 90 min with 50 μg/mL recombinant protein (LigBC, Lp25 or GroEL). Subsequently, the membrane was washed five times with PBS-T for 5 min and incubated at room temperature for 60 min with mouse antibody recognizing the respective recombinant protein of the first incubation at a 1:5000 dilution. After three washes with PBS-T, the membrane was incubated for 60 min with a peroxidase conjugated secondary antibody immunoglobulin G (IgG) at a 1:5000 dilution at room temperature. The membrane was washed three times with PBS-T after the incubation. Enhanced chemioluminescense (West Pico; Pierce, Thermo Fisher Scientific Inc., Rockford, I.L.) detected the positive signal.

### Elastin binding

The ability of recombinant proteins to bind to insoluble elastin was performed as described by Yang et al. [75] with minor modifications. Different quantities of elastin (0.5, 1, 5 and 10 mg) were mixed with 40 μg of each recombinant protein in a final volume of 400 μl of PBS. The samples were incubated for 60 min at 4 °C with 250 rpm stirring. Samples were centrifuged for 20 min at 18,000 x*g,* 4 °C. The supernatants, containing unbound proteins, were collected for SDS-PAGE analysis and the pellets were washed three times with 400 μL of PBS and resuspended with the same volume of 10% SDS and boiled for 10 min to release bound protein. The samples were analyzed by 12% SDS-PAGE gel. BSA was used as a negative control in place of the proteins in the incubation. LigBC was used as a positive control.

### Cell binding assays

Evaluation of cell binding activity of proteins was performed as previously described [41]. Briefly, VERO cells (monkey kidney epithelial cells – *Cercopithecus aethiops*) were cultured into a 96-well plate (Costar®) using 10% FBS-DMEM supplemented with 5 mM L-glutamine at 37 °C in humidified incubator with 5% CO_2_. Confluent cells were washed with PBS and fixed with 0.1% formaldehyde in PBS for 10 min at 37 °C. The formaldehyde was removed and the cells were washed with PBS and blocked for 1 h with 2% BSA. The cells were incubated with 5 or 10 μg/mL of each recombinant protein diluted in PBS for 2 h at 37 °C. After, three washes with PBS, the protein binding was detected with mouse anti-recombinant proteins at a 1:5000 dilution followed by peroxidase-conjugated anti-mouse IgG at a 1:5000 dilution. The substrate reaction was performed with ortho-phenylenediamine dihydrochloride and absorbance was measured at 492 nm.

### Cytokine secretion assay

J774A.1 cells (1 × 10^6^ cells/mL) were cultured overnight using 10% FBS-MEM into a 96-well plate (Costar®) at 37 °C in humidified incubator with 5% CO_2_. The cells were stimulated with 1, 5 and 10 μg/mL of purified GroEL and LPS (50 ng/mL), both treated and untreated with 50 μg/mL of polymyxin B and/or 50 μg/mL of proteinase K. After 20 h of incubation at 37 °C, the concentrations of IL-2, IL-4, IL-6, IFN-γ, TNF-α, IL-17A, and IL-10, in culture media were measured by flow cytometry using the BD Cytometric Bead Array (CBA) Mouse Th1/Th2/Th17 Cytokine Kit (BD Biosciences®), following the manufacturer’s instructions and the flow cytometer BD FACSCanto II (BD Biosciences®).

### Isolation of whole-cell and culture supernatant fractions

*L. interrogans* serovar Copenhageni strain Fiocruz L1–130 (ATCC BAA-1198) was cultivated at 29 °C under aerobic conditions in liquid EMJH medium for 7 days. Cells were harvested by centrifugation at 5500 x g for 30 min at 20 °C and gently washed three times with PBS. Cellular pellets were suspended to a final volume of PBS to obtain 1 × 10^9^ cells/mL, counted by the Petroff Hauser chamber (Fisher Scientific), using a dark-field microscopy. The bacterial suspensions were incubated at 37 °C for 5 h to enable the protein secretion, as previously described [39]. The samples were centrifuged at 10,000 x g for 30 min at 4 °C and the supernatants were collected and subjected to filtration using 0.2 μm filter unit (Millipore). The cellular pellet (whole-cell fraction) was resuspended in PBS. All fractions were subjected to 10% SDS-PAGE and transferred to nitrocellulose membranes for immunological analysis.

### Isolation of Outer Membrane Proteins (OMPs)

Leptospires were cultured and the cellular pellet were obtained as outlined above. OMPs from cellular pellet were extracted using Triton X-114 as previously described [76]. Two fractions were obtained: (A) the aqueous phase with periplasmatic proteins and (B) the Triton X-114 phase that corresponds to OMPs. All fractions were analyzed using immunoblots with antisera against recombinant proteins.

### Proteinase K accessibility assay

Leptospires were cultured as described above and centrifuged at 2.000×*g* 30 min. The sediment was washed twice with PBS and 5 mM MgCl_2_. Cells were gently suspended in the same buffer (final concentration of 2 × 10^9^ cells/mL). Analyses of the GroEL localization in the leptospiral surface was performed as described by Pinne & Haake [77], using different concentrations (from 0 to 200 μg/mL) of proteinase K enzyme.

### Immunoblot analysis

Whole-cell, secreted protein, OMPs fractions and samples of proteinase K treatments were transferred to nitrocellulose membranes and probed with anti-GroEL, anti-LipL31 (cytoplasmatic control protein), and anti-LigA (surface membrane control protein) diluted 1:500, followed by peroxidase-conjugated anti-mouse IgG at a 1:5000 dilution. The immunoreactive proteins were identified by Supersignal West Pico Chemiluminescent Substrate kit (Thermo Fisher Scientific, Boston, MA, USA).

### Statistical analysis

Student’s unpaired *t*-test was used to determine statistical significance of values obtained. Values of *p* < 0.05 were considered statistically significant.

## Supplementary Information

**Additional file 1: Figure S1.** Multiple sequence alignment performed using Clustal Omega program. a) Chaperonins group I: alignment of amino acid sequences of GroEL from human mitochondria (PDB: 4PJ1_A), *L.interrogans* (AAS69936.1), *Escherichia coli* (EFS2935563.1), and *Thermus thermophilus* (WP_011174077.1). The initial sequence of mitocrondial GroEL, residues 1–26 (MGSHHHHHHHHGSDYDIPTTENLYFQ), was excluded from the figure for better representation. b) Chaperonins group II: Alignment of amino acid sequences of GroEL from human TCP1 (T-complex protein 1) (NP_110379.2), Drosophila TCP1 (AAA28927.1), *Escherichia coli* (EFS2935563.1), *L.interrogans* (AAS69936.1), and *Thermus thermophilus* (WP_011174077.1). The equatorial domain (subdomains E1 and E2) is indicated in blue; intermediate domain (subdomains I1 and I2) in red and apical domain in green. The highly conserved motifs were underlined. (*) asterisk indicates identical residues (fully conserved) in all sequences; (:) colon shows conservation between residues with similar properties (strong conservation), and (.) period represents conservation between residues with weakly similar properties. Protein accession sequences are available in http://www.ncbi.nlm.nih.gov/protein/. **Figure S2.** Multiple sequence alignment performed using Clustal Omega program of amino acid sequences of GroEL from *L. interrogans* Copenhageni strain Fiocruz L1–130 and the most conserved sequences from each leptospiral taxid in the NCBI databases, as described in Table S1. Strains highlighted in yellow are saprophytic. The equatorial domain (subdomains E1 and E2) is indicated in blue box, intermediate domain (subdomains I1 and I2) in red box and apical domain in green box. The conserved motifs were underlined. (*) asterisk indicates identical residues (fully conserved) in all sequences; (:) colon shows conservation between residues with similar proprieties (strong conservation), and (.) period and gray shaded amino acids represent conservation between residues with weakly similar properties. Yellow shaded amino acids are divergent residues. **Figure S3**. a) Analysis of purified proteins. b) Proteinase K susceptibility of recombinant proteins, which were incubated with two different concentrations of proteinase K (1 and 100 μg/mL). Protein bands were visualized by Coomassie blue staining on 12% SDS-PAGE. M: molecular weight protein markers. **Figure S4.** Full-length Western blots of Fig. 2. Subcellular localization of the GroEL protein. *L. interrogans* serovar Copenhageni strain Fiocruz L1–130 was cultivated at 29 °C and in conditions that mimics the host environment, with shift temperatures from 29 to 37 °C for 5 h and in osmolarity of 300 mOsm. a) Whole-cell lysates (W) and cell culture supernatant fractions (S) were analyzed by immunoblotting using anti-GroEL and anti-LipL31 (positive control of cytoplasmatic protein) antisera. b) Whole cell (W), Triton X-114 fractions (A and D), and culture supernatant fraction (S) were analyzed by immunoblotting with anti-GroEL and anti-LipL31. c) Proteinase K accessibility assay. Intact leptospires were incubated with different concentrations of proteinase K and processed for immunoblot analyses using antibodies against GroEL, LipL31 or LigA (positive control of outer membrane protein). Protein ladder used was the color prestained protein Standard, broad range (Biolabs). **Figure S5.** Full-length Western Blot Overlay of Fig. 4. Collagen type I, collagen type IV, laminin, plasminogen, plasma fibronectin, fibrinogen, and BSA (negative control) were subjected to 4–20% gradient gel (SDS PAGE) under reducing conditions, transferred to nitrocellulose membranes and stained with Ponceau S (right). After incubation of membrane with recombinant proteins, the binding was detected with polyclonal antibodies recognizing Lp25, LigBC and GroEL. Chains of host proteins are indicated by γ (400 kDa), β (270 kDa), α2 (130 kDa), α1 (115 kDa) of collagen type I; α1 (140 kDa), α2 (160 kDa) of collagen type IV; C1 (400 kDa) and C2 (200 kDa) of laminin; α (220 kDa) and β (212 kDa) of plasma fibronectin; α (63.5 kDa), β (56 kDa), γ (47 kDa) of fibrinogen; plasminogen and BSA with 70 kDa and 66 kDa, respectively. Lp25 and LigBC were used as negative and positive binding controls, respectively. Protein ladder used was the prestained full-range rainbow RPN800E (Amersham). **Table S1.** Name, accession taxid numbers, and sequence identity of GroEL.

## Data Availability

All data generated or analyzed during this study are included in this published article. The accession numbers of representative sequences of chaperonins are AAS69936.1 (*L.interrogans*), EFS2935563.1 (*E.coli*), WP_011174077.1 (*T. thermophilus*), NP_110379.2 (human TCP1), AAA28927.1 (*Drosophila* TCP1) found in https://www.ncbi.nlm.nih.gov/. The data generated by bioinformatic analysis and the sequences used in the multiple alignments are available in the Butantan Institute Repository [https://repositorio.butantan.gov.br/handle/butantan/3635].
