# Supplementary material for: GroEL protein of the Leptospira spp. interacts with host proteins and induces cytokines secretion on macrophages
Source: BMC Microbiol. 2021 Mar 31;21:99. doi: 10.1186/s12866-021-02162-w (PMC8011160; doi:10.1186/s12866-021-02162-w)
Supplement: Supplementary file 1 — Additional file 1: Figure S1. Multiple sequence alignment performed using Clustal Omega program. a) Chaperonins group I: alignment of amino acid sequences of GroEL from human mitochondria (PDB: 4PJ1_A), L.interrogans (AAS69936.1), Escherichia coli (EFS2935563.1), and Thermus thermophilus (WP_011174077.1). The initial sequence of mitocrondial GroEL, residues 1–26 (MGSHHHHHHHHGSDYDIPTTENLYFQ), was excluded from the figure for better representation. b) Chaperonins group II: Alignment of amino acid sequences of GroEL from human TCP1 (T-complex protein 1) (NP_110379.2), Drosophila TCP1 (AAA28927.1), Escherichia coli (EFS2935563.1), L.interrogans (AAS69936.1), and Thermus thermophilus (WP_011174077.1). The equatorial domain (subdomains E1 and E2) is indicated in blue; intermediate domain (subdomains I1 and I2) in red and apical domain in green. The highly conserved motifs were underlined. (*) asterisk indicates identical residues (fully conserved) in all sequences; (:) colon shows conservation between residues with similar properties (strong conservation), and (.) period represents conservation between residues with weakly similar properties. Protein accession sequences are available in http://www.ncbi.nlm.nih.gov/protein/. Figure S2. Multiple sequence alignment performed using Clustal Omega program of amino acid sequences of GroEL from L. interrogans Copenhageni strain Fiocruz L1–130 and the most conserved sequences from each leptospiral taxid in the NCBI databases, as described in Table S1. Strains highlighted in yellow are saprophytic. The equatorial domain (subdomains E1 and E2) is indicated in blue box, intermediate domain (subdomains I1 and I2) in red box and apical domain in green box. The conserved motifs were underlined. (*) asterisk indicates identical residues (fully conserved) in all sequences; (:) colon shows conservation between residues with similar proprieties (strong conservation), and (.) period and gray shaded amino acids represent conservation between [file 12866_2021_2162_MOESM1_ESM.pdf]

Figure S1(a)

```
                                                        E1
Mitochondrial   GSAKDVKFGADARALMLQGVDLLADAVAVTMGPKGRTVIIEQSWGSPKVTKDGVTVAKSI  60
E.coli          MAAKDIRFGEDARARMVRGVNVLANAVKATLGPKGRNVVLEKSFGAPTITKDGVSVAKEI  60
Lepto           -MAKDIEYNETARRKLLEGVNKLANAVKVTLGPKGRNVVIDKKFGAPTITKDGVTVAKEI  59
Thermus         -MAKILVFDEAARRALERGVNAVANAVKVTLGPRGRNVVLEKKFGSPTITKDGVTVAKEV  59
                  ** : :.  **  : .**: :*:** .*:**:**.*::::.:*:*.:*****:***.:

Mitochondrial   DLKDKYKNIGAKLVQDVANNTNEEAGDGTTTATVLARSIAKEGFEKISKGANPVEIRRGV  120
E.coli          ELADKFENMGAQMVKEVASKTSDNAGDGTTTATVLAQALIREGMKAVAAGMNPMDLKRGI  120
Lepto           ELEDPLENMGAQMVKEVSTKTNDVAGDGTTTATILAQSIINEGLKNVTAGANPMSLKKGI  119
Thermus         ELEDHLENIGAQLLKEVASKTNDVAGDGTTTATVLAQAIVREGLKNVAAGANPLALKRGI  119
                :* *  :*:**:::::*:.:*.: *********:**::: .**:: :: * **: :::*:
                                                          I1
Mitochondrial   MLAVDAVIAELKKQSKPVTTPEEIAQVATISANGDKEIGNIISDAMKKVGRKGVITVKDG  180
E.coli          DKAVTSAVEELKKISKPCSTSKEIAQVGSISANSDTDIGELIAKAMDKVGKEGVITVEEG  180
Lepto           DKAVTAAVESIQKRAVKIENKKDIANVASISANNDNTIGNLIADAMDKVGKDGVITVEEA  179
Thermus         EKAVEAAVEKIKALAIPVEDRKAIEEVATISAN-DPEVGKLIADAMEKVGKEGIITVEES  178
                 ** :.: .::  :      : * :*.:**** *  :*::*:.**.***:.*:***::.
                                                          AD
Mitochondrial   KTLNDELEIIEGMKFDRGYISPYFINTSKGQKCEFQDAYVLLSEKKISSIQSIVPALEIA  240
E.coli          SGLENELDVVEGMQFDRGYLSPYFINNPQSMQAELEDPFILLHDKKISNVRDLLPILEGV  240
Lepto           KSIETTLDVVEGMQFDRGYISPYMVTDAESMVATLNDPFILIYDKKISSMKDLIHILEKV  239
Thermus         KSLETELKFVEGYQFDKGYISPYFVTNPETMEAVLEDAFILIVEKKVSNVRELLPILEQV  238
                . ::  *..:** :**:**:***::.  :   . ::* ::*: :**:*.::.::  ** .

Mitochondrial   NAHRKPLVIIAEDVDGEALSTLVLNRLKVGLQVVAVKAPGFGDNRKNQLKDMAIATGGAV  300
E.coli          AKAGKPLLIVAEDVEGEALATLVVNTIRGIVKVCAVKAPGFGDRRKAMLEDMAILTGGTV  300
Lepto           AQAGKPLVIISEEVEGEALATIVVNTLRKTISCVAVKAPGFGDRRKSMLEDIAILTGGQV  299
Thermus         AQTGKPLLIIAEDVEGEALATLVVNKLRGTLSVAAVKAPGFGDRRKEMLKDIAAVTGGTV  298
                    ***:*::*:*:****:*:*:* ::  :.  *********.**  *:*:*  *** *

Mitochondrial   NAHRKPLVIIAEDVDGEALSTLVLNRLKVGLQVVAVKAPGFGDNRKNQLKDMAIATGGAV  360
E.coli          ISE-EVGLSLEKATINDLGRAKKVQVSKENTTIIDGAGDTADIEARIKQIKAQIEETTSD  359
Lepto           ISE-DLGMKLENTTLQMLGRANKVTVDKENTTIIEGKGQTKEIQGRIGQIKKQIEDTTSE  358
Thermus         ISE-ELGFKLENATLSMLGRAERVRITKDETTIVGGKGKKEDIEARINGIKKELETTDSE  357
                :.*  : :.**..    **:. .* : *::: :: * *.. :*: **  *  ::: * *:
                                                          I2
Mitochondrial   YEKEKLNERLAKLSDGVAVLKVGGTSDVEVNEKKDRVTDALNATRAAVEEGIVLGGGCAL  420
E.coli          YDREKLQERVAKLAGGVAVIKVGAATEVEMKEKKARVEDALHATRAAVEEGIVPGGGVAL  419
Lepto           YDREKLQERLAKLAGGVAVIHVGAATEVEMKEKKARVEDALSATRAAVEEGIVPGGGLTL  418
Thermus         YAREKLQERLAKLAGGVAVIRVGAATETELKEKKHRFEDALNATRAAVEEGIVPGGGVTL  417
                * :***:**:***:.****::**.:::.*::*** *. *** *********** *** :*
                                                          E2
Mitochondrial   LRCIPALDSLT-PANEDQKIGIEIIKRTLKIPAMTIAKNAGVEGSLIVEKIMQ--SSSEV  477
E.coli          IRAKAAIAELK-GANEDQNHGIAIALRAMEAPLREIVTNAGDEPSVVLNRVAE--GTGAF  476
Lepto           LKAQEAVGSL--KLDGDEATGAKIIFRALEEPIRMITSNAGLEGSVIVEHAKA--KKGNE  474
Thermus         LRAISAVEELIKKLEGDEATGAKIVRRALEEPARQIAENAGYEGSVIVQQILAETKNPRY  477
                ::.  *: .*    : *:  *  *  *::: *   *. *** * *:::::      .

Mitochondrial   GYDAMAGDFVNMVEKGIIDPTKVVRTALLDAAGVASLLTTAEVVVTEIPKEEKDP-----  528
E.coli          GYNAANGEFGDMIEFGILDPTKVTRSALQNAASIAGLMITTEAMVAEAPKKEEPAA----  532
Lepto           GFNALTMVWEDMIQAGVVDPAKVVRSALQNAASIGSMILTTEVTITDKPDKDAPNPMAGM  534
Thermus         GFNAATGEFVDMVEAGIVDPAKVTRSALQNAASIGALILTTEAVVAEKPEKKESTPASA-  536
                *::*    : :*:: *::**:**.*:** :**.:..:: *:*. ::: *.:

Mitochondrial   -------------    547
E.coli          PGGGMGGMGGMDF    545
Lepto           GGGGMGGMGGMM-    546
Thermus         GAGDMDF------    543
```

Figure S1(b)

```
TCP1H      ---MEGPLSVFGDRSTGETIRSQNVMAAASIANIVKSSLGPVGLDKMLVDDIGDVTITND   57
TCP1D      MSTLASPLSIAGTRQSRRIVRTQNVMAALSISNIVKSSLGPVGLDKMLVDDIGDVTVTND   60
E.coli     ---MAAKDIRFGEDARARMVRG-----VNVLANAVKATLGPKGRNVVLEKSFGAPTITKD   52
Lepto      ----MAKDIEYNETARRKLLEG-----VNKLANAVKVTLGPKGRNVVIDKKFGAPTITKD   51
Thermus    ----MAKILVFDEAARRALERG-----VNAVANAVKVTLGPRGRNVVLEKKFGSPTITKD   51
                .     .        .      .  ::* ** :*** * : :: ..:*  *:*:*

TCP1H      GATILKLLEVEHPA----AKVLCELADLQDKEVGDGTTSVVIIAAELLKNADELVKQKIH   113
TCP1D      GATILRLLEVEHPA----AKVLVELAQLQDEEVGDGTTSVVILAAELLKNADELVKQKIH   116
E.coli     GVSVAKEIELADKFENMGAQMVKEVASKTSDNAGDGTTTATVLAQALIREGMKAVAAGMN   112
Lepto      GVTVAKEIELEDPLENMGAQMVKEVSTKTNDVAGDGTTTATILAQSIINEGLKNVTAGAN   111
Thermus    GVTVAKEVELEDHLENIGAQLLKEVASKTNDVAGDGTTTATVLAQAIVREGLKNVAAGAN   111
           *.:: : :*: .      *::: *::   .. .*****:..::*  ::.:. : *    :

TCP1H      PTSVISGYRLACKEAVRYINENLI---------------------------VNTDELGRD   146
TCP1D      PTSIISGYRIACKEACKYISEHLT---------------------------APVDELGRD   149
E.coli     PMDLKRGIDKAVTSAVEELKKISKPCSTSKEIAQVGSISANSDTDIGELIAKAMDKVGKE   172
Lepto      PMSLKKGIDKAVTAAVESIQKRAVKIENKKDIANVASISANNDNTIGNLIADAMDKVGKD   171
Thermus    PLALKRGIEKAVEAAVEKIKALAIPVEDRKAIEEVATISAN-DPEVGKLIADAMEKVGKE   170
           *  :  *   *   * . :.                                  :::*::

TCP1H      CLINAAKTSMSSKIIGINGDFFANMVVDAVLAIKYTDIRGQPRYPVNSVNILKAHGRSQM   206
TCP1D      SLINIAKTSMSSKIIGADAEFFSAMVVDAAQSVKITDPRGQAVYSIKAVNVLKAHGKSAR   209
E.coli     GVITVEEGS----GLENELDVVEGMQF---------------------------------   195
Lepto      GVITVEEAK----SIETTLDVVEGMQF---------------------------------   194
Thermus    GIITVEESK----SLETELKFVEGYQF---------------------------------   193
           :*.  :  .     :    ...    .

TCP1H      ESMLISGYALNCVVGSQGMPKRIVNAKIACLDFSLQKTKMKLGVQVVITDPEKLDQIRQR   266
TCP1D      ESVLIPGYALNCTIASQQMPKKIVNAKIACHDFSLQKTKMKMGVQVLINDPDKLEAIRAR   269
E.coli     DRGYLSPYFINN---PQSMQAELEDPFILLHDKKISN------VRDLLPILEG-------   239
Lepto      DRGYISPYMVTD---AESMVATLNDPFILIYDKKISS------MKDLIHILEK-------   238
Thermus    DKGYISPYFVTN---PETMEAVLEDAFILIVEKKVSN------VRELLPILEQ-------   237
           :   :  * :.     : *   : :  *   : .:..      :: ::    :

TCP1H      ESDITKERIQKILATGANVILTTGGIDDMCLKY-------------FVEAGAMAVRRVLK   313
TCP1D      ELDITKERINMILGTGVNVVLVSGGVDDLCMKY-------------FVEAGAMAVRRVKK   316
E.coli     -----------VAKAGKPLLIVAEDVEGEALATLVVNTIRGIVKVCAVKAPG--FGDRRK   286
Lepto      -----------VAQAGKPLVIISEEVEGEALATIVVNTLRKTISCVAVKAPG--FGDRRK   285
Thermus    -----------VAQTGKPLLIIAEDVEGEALATLVVNKLRGTLSVAAVKAPG--FGDRRK   284
                      :  :*  ::: :  ::. .:                *:* .  .    *

TCP1H      RDLKRIAKASGATILSTLANLEGEETFEAAMLGQAEEVVQERICDDELILIKNT------   367
TCP1D      SDLKIIAKATGAAFITSLTNMDGEESFDASMVGEAAEVAQERICDDELILIKGT------   370
E.coli     AMLEDMAILTGGTVISEEVGLSLE-KATINDLGRAKKVVQS---KENTTIIDGAGDTADI   342
Lepto      SMLEDIAILTGGQVISEDLGMKLE-NTTLQMLGRANKVTVD---KENTTIIEGKGQTKEI   341
Thermus    EMLKDIAAVTGGTVISEELGFKLE-NATLSMLGRAERVRIT---KDETTIVGGKGKKEDI   340
             *: :*  :*. .::   .:. * .     :*.* .*      .::  :: .

TCP1H      ----------------------------K-ARTSASIILRGANDFMCDEMERSLHDALCV   398
TCP1D      ----------------------------K-ARAAASIILRGPNDFYCDEMERSVHDALCV   401
E.coli     EARIKQIKAQIEETTSDYDREKLQERVAKLAGGVAVIKVGAATEVEMKEKKARVEDALHA   402
Lepto      QGRIGQIKKQIEDTTSEYDREKLQERLAKLAGGVAVIHVGAATEVEMKEKKARVEDALSA   401
Thermus    EARINGIKKELETTDSEYAREKLQERLAKLAGGVAVIRVGAATETELKEKKHRFEDALNA   400
                                       * *   * * : . .:   .* :  ..*** .

TCP1H      VKRVLESKSVVPGGGAVEAALSIYLENYATSMGSREQLAIAEFARSLLVIPNTLAVNAAQ   458
TCP1D      VKRVLESKKVVRGGGCVEAALSIYLENFATSLASREQLAIAEFAKSLLVIPKTLSVNAAK   461
E.coli     TCAAV-EEGIVPGGGVALIRAKAAIAELK-GANEDQNHGIAIALRAMEAPLREIVTNAGD   460
Lepto      TRAAV-EEGIVPGGGLTLLKAQEAVGSL--KLDGDEATGAKIIFRALEEPIRMITSNAGL   458
Thermus    TRAAV-EEGIVPGGGVTLLRAISAVEELIKKLEGDEATGAKIVRRALEEPARQIAENAGY   459
           .  .: .: :* *** .       : .        :  .     :::    . :  **.

TCP1H      DSTDLVAKLRAFHNEAQVNPERKNLKWIGLDLSNGKPRDNKQAGVFEPTIVKVKSLKFAT   518
TCP1D      DATDLVAKLRSYHNSSQTKPERSDLKWTGLDLIEGVVRDNKKAGVLEPAMSKIKSLKFAT   521
E.coli     EPSVVLNRVA----------E--GTGAFGYNAANGEFGDMIEFGILDPTKVTRSALQNAA   508
Lepto      EGSVIVEHAK----------A--KKGNEGFNALTMVWEDMIQAGVVDPAKVVRSALQNAA   506
Thermus    EGSVIVQQIL----------AETKNPRYGFNAATGEFVDMVEAGIVDPAKVTRSALQNAA   509
           : : :: :                    * :       *  : *:.:*:    .:*: *:

TCP1H      EAAITILRIDDLIKLHPESKDDKHGSYEDAVHSGALND------   556
TCP1D      EAAITILRIDDMIKLNPEDKSGK--SYADACAAGELDG------   557
E.coli     SIAGLMITTEAMVAEAPKKEEPAA-------PGGGMGGMGGMDF   545
Lepto      SIGSMILTTEVTITDKPDKDAPNPMA---GMGGGGMGGMGGMM-   546
Thermus    SIGALILTTEAVVAEKPEKKESTPAS---A-GAGDMDF------   543
```

Table S1. Name, accession taxid numbers, and sequence identity of GroEL

**Name**		GroEL / Cpn60 / Hsp60
**Gene ID** [a]		LIC11335
**Protein accession no.**[b]		AAS69936.1
**% Amino acid sequence identity** [c]	**1-** *L.interrogans* (taxid:173)	97.28 - 100
	**2-** *L.kirschneri* (taxid:29507)	98.37 - 100
	**3-** *L. noguchii* (taxid:28182)	99.08 - 99.45
	**4-** *L.santarosai* (taxid:28183)	96.70 - 97.25
	**5-** *L. weilii (*taxid:28184)	96.21 – 96.52
	**6-** *L. alexanderi* (taxid:100053)	96.52
	**7-** *L. borgpetersenii* (taxid:174)	96.34 – 96.52
	**8-** *L. wolffii* (taxid:409998)	91.06 - 91.25
	**9-** *L. alstoni* (taxid:28452)	96.70
	**10-** *L. kmetyi (taxid:408139)*	97.62
	**11-** *L. biflexa* (taxid:172)	81.08 – 86.26
	**12-** *L. meyeri* (taxid:29508)	86.26
	**13-** *L. wolbachii* (taxid:29511)	86.07
	**14-** *L. vanthielii* (taxid:293085)	85.88
	**15-** *L. terpstrae* (taxid:293075)	87.02
	**16-** *L. yanagawae* (taxid:293069)	86.26

[a] ID, identifier; LIC, *Leptospira interrogans* Copenhageni strain Fiocruz L1-130.
b http://www.ncbi.nlm.nih.gov/protein/.
[c] Data are percentage of identity between sequences of GroEL from *L. interrogans* Copenhageni strain Fiocruz L1-130 and other sequences from leptospiral taxid in the NCBI databases. These analyses were performed by BLASTp program (http://www.ncbi.nlm.nih.gov/blast/Blast.cgi). Only sequences, which have aligned to the query with E Value ≤ zero, were considered for analyses. Numbers 1-10 are pathogenic and 11-16 are saprophytic leptospira strains.

Figure S2

```
L.wolbachii          MAKTIEFDETARRKLLSGVNKLANAVKVTLGPKGRNVVIDKKFGAPTITKDGVTVAKEIE   60
L.vanthielii         MAKTIEFDETARRKLLSGVNKLANAVKVTLGPKGRNVVIDKKFGSPTITKDGVTVAKEIE   60
L.terpstrae          MAKTIEFDETARRKLLSGVNKLANAVKVTLGPKGRNVVIDKKFGAPTITKDGVTVAKEIE   60
L.meyeri             MAKTIEFDETARRKLLSGVNKLANAVKVTLGPKGRNVVIDKKFGSPTITKDGVTVAKEIE   60
L.biflexa            MAKTIEFDETARRKLLSGVNKLANAVKVTLGPKGRNVVIDKKFGSPTITKDGVTVAKEIE   60
L.yanagawae          MAKTIEFDESARRKLLSGVNKLANAVKVTLGPKGRNVVIDKKFGSPTITKDGVTVAKEIE   60
L.wolffii            MAKVIEYDETARRKLLEGVNKLANAVKVTLGPKGRNVVIDKKFGSPTITKDGVTVAKEIE   60
L.kmetyi             MAKDIEYNETARRKLLEGVNKLANAVKVTLGPKGRNVVIDKKFGAPTITKDGVTVAKEIE   60
L.santarosai         MAKDIEYNETARRKLLEGVNKLANAVKVTLGPKGRNVVIDKKFGAATITKDGVTVAKEIE   60
L.interrogans        MAKDIEYNETARRKLLEGVNKLANAVKVTLGPKGRNVVIDKKFGAPTITKDGVTVAKEIE   60
L.kirschneri         MAKDIEYNETARRKLLEGVNKLANAVKVTLGPKGRNVVIDKKFGAPTITKDGVTVAKEIE   60
L.noguchii           MAKDIEYNETARRKLLEGVNKLANAVKVTLGPKGRNVVIDKKFGAPTITKDGVTVAKEIE   60
L.alstonii           MAKDIEYNETARRKLLEGVNKLANAVKVTLGPKGRNVVIDKKFGAPTITKDGVTVAKEIE   60
L.borgpetersenii     MAKDIEYNETARRKLLEGVNKLANAVKVTLGPKGRNVVIDKKFGAPTITKDGVTVAKEIE   60
L.weilii             MAKDIEYNETARRKLLEGVNKLANAVKVTLGPKGRNVVIDKKFGAPTITKDGVTVAKEIE   60
L.alexanderi         MAKDIEYNETARRKLLEGVNKLANAVKVTLGPKGRNVVIDKKFGAPTITKDGVTVAKEIE   60
                     *** **::*:******.***************************: **************

L.wolbachii          LEDAIENMGAQMVKEVSTRTNDIAGDGTTTATILAQAIINEGLKNVTAGANPMALKHGID   120
L.vanthielii         LEDAIENMGAQMVKEVSTRTNDIAGDGTTTATILAQAIINEGLKNVTAGANPMALKHGID   120
L.terpstrae          LEDPIENMGAQMVKEVSTKTNDIAGDGTTTATILAQAIINEGLKNVTAGANPMALKHGID   120
L.meyeri             LEDAIENMGAQMVKEVSTKTNDIAGDGTTTATILAQAIINEGLKNVTAGANPMALKHGID   120
L.biflexa            LEDAIENMGAQMVKEVSTKTNDIAGDGTTTATILAQAIINEGLKNVTAGANPMALKHGID   120
L.yanagawae          LEDAIENMGAQMVKEVSTKTNDIAGDGTTTATILAQAIINEGLKNVTAGANPMALKHGID   120
L.wolffii            LEDAIENMGAQMVKEVSTKTNDVAGDGTTTATILAQSIVNEGLKNVTAGANPMALKHGID   120
L.kmetyi             LEDPLENMGAQMVKEVSTKTNDVAGDGTTTATILAQSIINEGLKNVTAGANPMSLKRGID   120
L.santarosai         LDDPLENMGAQMVKEVSTKTNDVAGDGTTTATILAQSIINEGLKNVTAGANPMSLKRGID   120
L.interrogans        LEDPLENMGAQMVKEVSTKTNDVAGDGTTTATILAQSIINEGLKNVTAGANPMSLKKGID   120
L.kirschneri         LEDPLENMGAQMVKEVSTKTNDVAGDGTTTATILAQSIINEGLKNVTAGANPMSLKRGID   120
L.noguchii           LEDPLENMGAQMVKEVSTKTNDVAGDGTTTATILAQSIINEGLKNVTAGANPMSLKRGID   120
L.alstonii           LEDPLENMGAQMVKEVSTKTNDVAGDGTTTATILAQSIINEGLKNVTAGANPMSLKRGID   120
L.borgpetersenii     LEDALENMGAQMVKEVSTKTNDVAGDGTTTATILAQSIINEGLKNVTAGANPMSLKRGID   120
L.weilii             LEDALENMGAQMVKEVSTKTNDVAGDGTTTATILAQSIINEGLKNVTAGANPMSLKRGID   120
L.alexanderi         LEDPLENMGAQMVKEVSTKTNDVAGDGTTTATILAQSIINEGLKNVTAGANPMSLKRGID   120
                     *:*  :*************:***:*************:*:**************:**:***

L.wolbachii          KAVVVAVEEIKKHAIKINSKAEYANVATISANNDPEIGNLIAQAFDKVGKEGVITVDEAK   180
L.vanthielii         KAVVVAVEEIKKHAIKINSKAEYANVATISANNDPEIGNLIAQAFDKVGKEGVITVDEAK   180
L.terpstrae          KAVLAAVEEIKKHAIKINSKAEYANVATISANNDPEIGNLIAQAFDKVGKEGVITVDEAK   180
L.meyeri             KAVLSAVEEIKKHAIKINSKAEYANVATISANNDPEIGNLIAQAFDKVGKEGVITVDEAK   180
L.biflexa            KAVVVAVEEIKKHAIKINSKAEYANVATISANNDPEIGNLIAQAFDKVGKEGVITVDEAK   180
L.yanagawae          KAVLAAVEEIKKHAIKINSKAEYANVATISANNDPEIGNLIAQAFDKVGKEGVITVDEAK   180
L.wolffii            KAVNAAVESIKKRSVKIENKKDIANVATISANNDKDIGNLIADAMDKVGKDGVITVEEAK   180
L.kmetyi             KAVSAAVESIQKRAVKIENKKDIANVATISANNDTTIGNLIADAMDKVGKDGVITVEEAK   180
L.santarosai         KAVTAAVEHIQKKAVKIENKKDIANVATISANNDDTIGNLIADAMDKVGKDGVITVEEAK   180
L.interrogans        KAVTAAVESIQKRAVKIENKKDIANVASISANNDNTIGNLIADAMDKVGKDGVITVEEAK   180
L.kirschneri         KAVTAAVESIQKRAVKIENKKDIANVASISANNDNTIGNLIADAMDKVGKDGVITVEEAK   180
L.noguchii           KAVTAAVESIQKRAVKIENKKDIANVAAISANNDNTIGNLIADAMDKVGKDGVITVEEAK   180
L.alstonii           KAVTAAVESIQKRAVKIENKKDIANVASISANNDNTIGTLIADAMDKVGKDGVITVEEAK   180
L.borgpetersenii     KAVTAAVESIRKRAVKIENKKDIANVASISANNDDTIGNLIADAMDKVGKDGVITVEEAK   180
L.weilii             KAVTAAVESIRKRAVKIENKKDIANVASISANNDDTIGTLIADAMDKVGKDGVITVEEAK   180
L.alexanderi         KAVTAAVESIRKRAVKIENKKDIANVASISANNDDTIGTLIADAMDKVGKDGVITVEEAK   180
                     ***   *** *:*:::**:.* : ****:******  **.***:*:*****:*****:***

L.wolbachii          SIETTLDIVEGMQFDRGYISPYMVTDPEAMVATFNDPFILIYDKKIASMKDLLPVLEKIA   240
L.vanthielii         SIETTLDIVEGMQFDRGYISPYMVTDPEAMVATFNDPFILIYDKKIASMKDLLPVLEKIA   240
L.terpstrae          SIETTLDIVEGMQFDRGYVSPYMVTDPEAMIATFNDPFILIYDKKIASMKDLLPVLEKIA   240
L.meyeri             SIETTLDIVEGMQFDRGYVSPYMVTDPEAMIATFNDPFILIYDKKIASMKDLLPVLEKIA   240
L.biflexa            SIETTLDIVEGMQFDRGYVSPYMVTDPEAMIATFNDPFILIYDKKIASMKDLLPVLEKIA   240
L.yanagawae          SIETTLDIVEGMQFDRGYVSPYMVTDPEAMIATFNDPFILIYDKKIASMKDLLPVLEKIA   240
L.wolffii            SIETTLDVVEGMQFDRGYVSPYMVTDPEAMIATLSDPYILIYDKKISSMRDLLPVLEKVA   240
L.kmetyi             SIETTLDVVEGMQFDRGYISPYMVTDPEAMTATLNDPFILIYDKKIASMKDLIHVLEKVA   240
L.santarosai         SIETTLDVVEGMQFDRGYISPYMVTDPESMVATLSDPYILIYDKKISSMKDLIHILEKVA   240
L.interrogans        SIETTLDVVEGMQFDRGYISPYMVTDAESMVATLNDPFILIYDKKISSMKDLIHILEKVA   240
L.kirschneri         SIETTLDVVEGMQFDRGYISPYMVTDAESMVATLNDPFILIYDKKISSMKDLIHILEKVA   240
L.noguchii           SIETTLDVVEGMQFDRGYISPYMVTDAESMVATLNDPFILIYDKKISSMKDLIHILEKVA   240
L.alstonii           SIETTLDVVEGMQFDRGYISPYMVTDAEAMIATLNDPFILIYDKKISSMKDLIHILEKVA   240
L.borgpetersenii     SIETTLDVVEGMQFDRGYISPYMVTDAESMVATLSDPYILIYDKKISSMKDLIHILEKVA   240
L.weilii             SIETTLDVVEGMQFDRGYISPYMVTDAESMVATLSDPYILIYDKKISSMKDLIHILEKVA   240
L.alexanderi         SIETTLDVVEGMQFDRGYISPYMVTDAESMVATLSDPYILIYDKKIASMKDLIHILEKVA   240
                     *******:*********:******* *:* **:.**:********:**:**: :***:*

L.wolbachii          QAGRPLVIIAEEVEGEALATIVVNTLRKTIQCVAVKAPGFGDRRKAMLEDIAVLTAGQVI   300
L.vanthielii         QAGRPLVIIAEEVEGEALATIVVNTLRKTIQCVAVKAPGFGDRRKAMLEDIAVLTAGQVI   300
L.terpstrae          QAGRPLVIIAEEVEGEALATIVVNTLRKTIQCVAVKAPGFGDRRKSMLEDIAILTGGQVI   300
L.meyeri             QAGRPLVIIAEEVEGEALATIVVNTLRKTIQCVAVKAPGFGDRRKAMLEDIAILTGGQVI   300
L.biflexa            QAGRPLVIIAEEVEGEALATIVVNTLRKTIQCVAVKAPGFGDRRKAMLEDIAILTGGQVI   300
L.yanagawae          QAGRPLVIIAEEVEGEALATIVVNTLRKTIQCVAVKAPGFGDRRKAMLEDIAILTGGQVI   300
L.wolffii            QAGRPLVIIAEEVEGEALATIVVNTLRKTISCVAVKAPGFGDRRKAMLEDIAILTGGQVI   300
L.kmetyi             QAGKPLVIISEEVEGEALATIVVNTLRKTISCVAVKAPGFGDRRKSMLEDIAILTGGQVI   300
L.santarosai         QAGKPLVIISEEVEGEALATIVVNTLRKTISCVAVKAPGFGDRRKAMLEDIAILTGGQVI   300
L.interrogans        QAGKPLVIISEEVEGEALATIVVNTLRKTISCVAVKAPGFGDRRKSMLEDIAILTGGQVI   300
L.kirschneri         QAGKPLVIISEEVEGEALATIVVNTLRKTISCVAVKAPGFGDRRKAMLEDIAILTGGQVI   300
L.noguchii           QAGKPLVIISEEVEGEALATIVVNTLRKTISCVAVKAPGFGDRRKAMLEDIAILTGGQVI   300
L.alstonii           QAGKPLVIISEEVEGEALATIVVNTLRKTISCVAVKAPGFGDRRKSMLEDIAILTGGQVI   300
L.borgpetersenii     QAGKPLVIISEEVEGEALATIVVNTLRKTISCVAVKAPGFGDRRKAMLEDIAILTGGQVI   300
L.weilii             QAGKPLVIISEEVEGEALATIVVNTLRKTISCVAVKAPGFGDRRKAMLEDIAILTGGQVI   300
L.alexanderi         QAGKPLVIISEEVEGEALATIVVNTLRKTISCVAVKAPGFGDRRKAMLEDIAILTGGQVI   300
                     ***:*****:*******************.*************:******:**.****

L.wolbachii          SEDLGMKLENAEVKMLGRAKKVVVDKENTTIIEGAGASKDIQGRVNQIKKQIEDTTSDYD   360
L.genomosp3          SEDLGMKLENAEVKMLGRAKKVVVDKENTTIIEGAGASKDIQGRVNQIKKQIEDTTSDYD   360
L.terpstrae          SEDLGMKLENADVKMLGRAKKVVVDKENTTIIEGAGASKDIQGRVNQIKKQIEDTTSDYD   360
L.meyeri             SEDLGMKLENADVKMLGRAKKVVVDKENTTIIEGAGASKDIQGRVNQIKKQIEDTTSDYD   360
```

```
L.biflexa           SEDLGMKLENADVKMLGRAKKVVVDKENTTIIEGAGASKDIQGRVNQIKKQIEDTTSDYD   360
L.yanagawae         SEDLGMKLENADVKMLGRAKKVVVDKENTTIIEGAGASKDIQGRVNQIKKQIEDTTSDYD   360
L.wolffii           SEDLGMKLENATVQQLGRAKKVTVDKENTTIIEGQGASKDIQGRVGQIKKQIDDTTSEYD   360
L.kmetyi            SEDLGMKLENTTLQMLGRANKVTVDKENTTIIEGKGQTKDIQGRIGQIKKQIEDTTSEYD   360
L.santarosai        SEDLGMKLENTTLQMLGRANKVTVDKENTTIIEGKGQTKEIQGRIGQIKKQIEDTSSEYD   360
L.interrogans       SEDLGMKLENTTLQMLGRANKVTVDKENTTIIEGKGQTKEIQGRIGQIKKQIEDTTSEYD   360
L.kirschneri        SEDLGMKLENTTLQMLGRANKVTVDKENTTIIEGKGQTKEIQGRIGQIKKQIEDTTSEYD   360
L.noguchii          SEDLGMKLENTTLQMLGRANKVTVDKENTTIIEGKGQTKEIQGRIGQIKKQIEDTTSEYD   360
L.alstonii          SEDLGMKLENATLQMLGRANKVTVDKENTTIIEGKGQTKDIQGRIGQIKKQIEDTTSEYD   360
L.borgpetersenii    SEDLGMKLENTTLQMLGRANKVTVDKENTTIIEGKGQTKEIQGRIGQIKKQIEDTTSEYD   360
L.weilii            SEDLGMKLENTTLQMLGRANKVTVDKENTTIIEGKGQTKEIQGRIGQIKKQIEDTTSEYD   360
L.alexanderi        SEDLGMKLENTTLQMLGRANKVTVDKENTTIIEGKGQTKEIQGRIGQIKKQIEDTTSEYD   360
                    **********: :: ****:**.*********** * :*:****:.******:**:*:**

L.wolbachii         REKLQERLAKLAGGVAVIHVGAATEVEMKEKKARVEDALSATRAAVEEGIVPGGGLTLLR   420
L.vanthielii        REKLQERLAKLAGGVAVIHVGAATEVEMKEKKARVEDALSATRAAVEEGIVPGGGLTLLR   420
L.terpstrae         REKLQERLAKLAGGVAVIHVGAATEVEMKEKKARVEDALSATRAAVEEGIVPGGGLTLLR   420
L.meyeri            REKLQERLAKLAGGVAVIHVGAATEVEMKEKKARVEDALSATRAAVEEGIVPGGGLTLLR   420
L.biflexa           REKLQERLAKLAGGVAVIHVGAATEVEMKEKKARVEDALSATRAAVEEGIVPGGGLTLLR   420
L.yanagawae         REKLQERLAKLAGGVAVIHVGAATEVEMKEKKARVEDALSATRAAVEEGIVPGGGLTLLR   420
L.wolffii           REKLQERLAKLAGGVAVIHVGAATEVEMKEKKARRVEDALATRAAVEEGIVPGGGLTLLK   420
L.kmetyi            REKLQERLAKLAGGVAVIHVGAATEVEMKEKKARVEDALSATRAAVEEGIVPGGGLTLLK   420
L.santarosai        REKLQERLAKLAGGVAVIHVGAATEVEMKEKKARVEDALSATRAAVEEGIVPGGGLTLLK   420
L.interrogans       REKLQERLAKLAGGVAVIHVGAATEVEMKEKKARVEDALSATRAAVEEGIVPGGGLTLLK   420
L.kirschneri        REKLQERLAKLAGGVAVIHVGAATEVEMKEKKARVEDALSATRAAVEEGIVPGGGLTLLK   420
L.noguchii          REKLQERLAKLAGGVAVIHVGAATEVEMKEKKARVEDALSATRAAVEEGIVPGGGLTLLK   420
L.alstonii          KEKLQERLAKLAGGVAVIHVGAATEVEMKEKKARVEDALSATRAAVEEGIVPGGGLTLLK   420
L.borgpetersenii    KEKLQERLAKLAGGVAVIHVGAATEVEMKEKKARVEDALSATRAAVEEGIVPGGGLTLLK   420
L.weilii            KEKLQERLAKLAGGVAVIHVGAATEVEMKEKKARVEDALSATRAAVEEGIVPGGGLTLLK   420
L.alexanderi        KEKLQERLAKLAGGVAVIHVGAATEVEMKEKKARVEDALSATRAAVEEGIVPGGGLTLLK   420
                    :**********************************************************:

L.wolbachii         AQDAVRALKLVGDEQTGVNIILRALEEPIRMITANAGLEGSVIVEQARAKKGNEGFNALT   480
L.vanthielii        AQDAVRALKLVGDEQTGVNIILRALEEPIRMITANAGLEGSVIVEQARAKKGNEGFNALT   480
L.terpstrae         AQDAVKALKLVGDEQTGANIILRALEEPIRMITSNAGLEGSVIVEQARARKGNEGFNALT   480
L.meyeri            AQDAVKALKLSGDEQTGANIILRALEEPIRMITSNAGLEGSVIVEQARGKKGNEGFNALT   480
L.biflexa           AQDAVKALKLVGDEQTGANIILRALEEPIRMITSNAGLEGSVIVEQARARKGNEGFNALT   480
L.yanagawae         AQDAVKALKLSGDEQTGANIILRALEEPIRMITSNAGLEGSVIVEQARARKGNEGFNALT   480
L.wolffii           AQEAVAALKLEGDEATGAKIIFRALEEPIRMITSNAGLEGSVIVEQAKGKKGNEGFNALT   480
L.kmetyi            AQEAVGGLKLEGDEATGAKIIFRALEEPIRMITSNAGLEGSVIVEHAKAKKGNEGFNALT   480
L.santarosai        AQEAVGSLKLEGDEATGAKIIFRALEEPIRMITSNAGLEGSVIVEHAKAKKGNEGFNALT   480
L.interrogans       AQEAVGSLKLDGDEATGAKIIFRALEEPIRMITSNAGLEGSVIVEHAKAKKGNEGFNALT   480
L.kirschneri        AQEAVGSLKLDGDEATGAKIIFRALEEPIRMITSNAGLEGSVIVEHAKAKKGNEGFNALT   480
L.noguchii          AQEAVGSLKLDGDEATGAKIIFRALEEPIRMITSNAGLEGSVIVEHAKAKKGNEGFNALT   480
L.alstonii          AQEAVSALKLEGDEATGAKIIFRALEEPIRMITNNAGLEGSVIVEHAKSKKGNEGFNALS   480
L.borgpetersenii    AQEAVAALKLEGDEATGAKIIFRSLEEPIRMITNNAGLEGSVIVEHAKTKKGNEGFNALS   480
L.weilii            AQEAVAALKLEGDEATGAKIIFRSLEEPIRMITNNAGLEGSVIVEHAKSKKGNEGFNALS   480
L.alexanderi        AQEAVAALKLEGDEATGAKIIFRSLEEPIRMITNNAGLEGSVIVEHAKAKKGNEGFNALS   480
                    **:** .*** *** **.:**:*:********* ***********:*: :*********:

L.wolbachii         MVWEDLIKAGVVDPAKVVRSALQNAASIGAMILTTEVTITDKPESKD-SGAGMAGMGGMG   539
L.vanthielii        MVWEDLIKAGVVDPAKVVRSALQNAASIGAMILTTEVTITDKPESKD-SGAGMAGMGGMG   539
L.terpstrae         MVWEDLIKAGVVDPAKVVRSALQNAASIGAMILTTEVTITDKPESKD-SGAGMAGMGGMG   539
L.meyeri            MVWEDLIKAGVVDPAKVVRSALQNAASIGAMILTTEVTITDKPEPKD-AGAGMAGMGGMG   539
L.biflexa           MVWEDLIKAGVVDPAKVVRSALQNAASIGAMILTTEVTITDKPEPKDTSGAGMGGMGGMG   540
L.yanagawae         MVWEDLIKAGVVDPAKVVRSALQNAASIGAMILTTEVTITDKPEPKDAAGAGMGGMGGMG   540
L.wolffii           MVWEDLLQAGVVDPAKVVRSALQNAASIGSMLLTTEVTITDKPEKDG---GGMPPMGGMG   537
L.kmetyi            MVWEDMIQAGVVDPAKVVRSALQNAASIGSMILTTEVTITDKPDKDA---PNPMAGMGG-   536
L.santarosai        MVWEDMIKAGVVDPAKVVRSALQNAASIGSMILTTEVTITDRPEKDA---PNPMAGMGGG   537
L.interrogans       MVWEDMIQAGVVDPAKVVRSALQNAASIGSMILTTEVTITDKPDKDA---PNPMAGMGGG   537
L.kirschneri        MVWEDMIQAGVVDPAKVVRSALQNAASIGSMILTTEVTITDKPDKDA---PNPMAGMGGG   537
L.noguchii          MVWEDMIQAGVVDPAKVVRSALQNAASIGSMILTTEVTITDKPDKDA---PNPMAGMGGG   537
L.alstonii          MVWEDLLAAGVVDPAKVVRSALQNAASIGSMILTTEVTITDKPEKDA---PNPMAGMGG-   536
L.borgpetersenii    MVWEDLVSAGVVDPAKVVRSALQNAASIGSMILTTEVTITDKPEKDA---PNPMAGMGGG   537
L.weilii            MVWEDLVAAGVVDPAKVVRSALQNAASIGSMILTTEVTITDKPEKDA---PNPMAGMGGG   537
L.alexanderi        MVWEDLVAAGVVDPAKVVRSALQNAASIGSMILTTEVTITDKPEKDA---PNPMAGMGGG   537
                    *****:: *********************:*:*********:*: .     .     * *

L.wolbachii         GMGGMGGMM      548
L.vanthielii        GMGGMGGMM      548
L.terpstrae         GMGGMGGMM      548
L.meyeri            GMGGMGGMM      548
L.biflexa           GMGGMGGMM      549
L.yanagawae         GMGGMGGMM      549
L.wolffii           GMGGMGGMM      546
L.kmetyi            GMGGMGGMM      545
L.santarosai        GMGGMGGMM      546
L.interrogans       GMGGMGGMM      546
L.kirschneri        GMGGMGGMM      546
L.noguchii          GMGGMGGMM      546
L.alstonii          GMGGMGGMM      545
L.borgpetersenii    GMGGMGGMM      546
L.weilii            GMGGMGGMM      546
L.alexanderi        GMGGMGGMM      546
                    *********
```

Figure S3

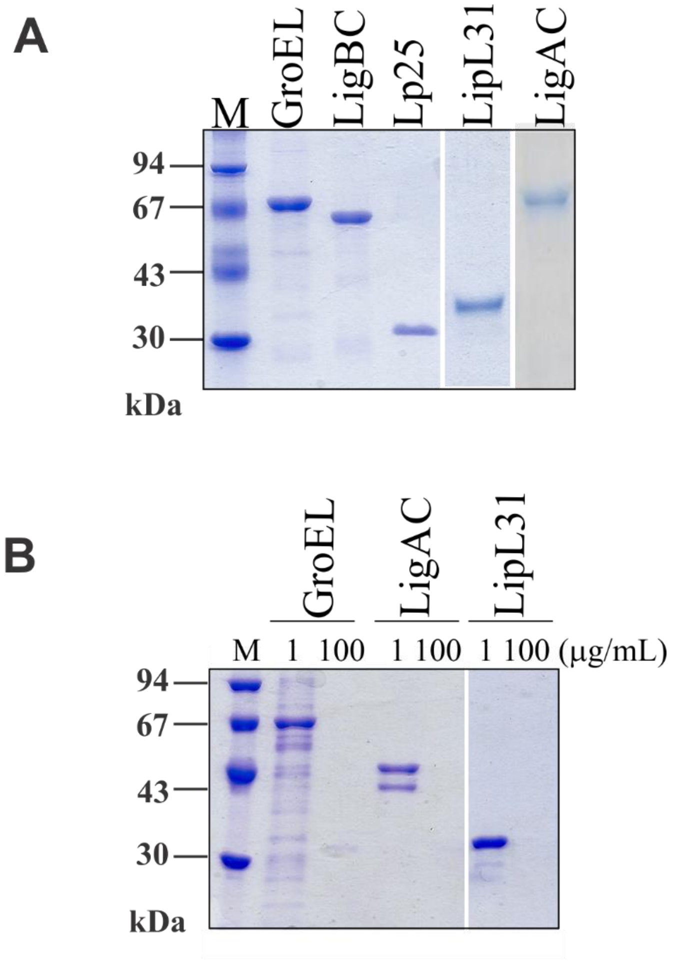
A
M
GroEL
LigBC
Lp25
LipL31
LigAC
94
67
43
30
kDa
B
GroEL
LigAC
LipL31
M
1
100
1
100
1
100
(µg/mL)
94
67
43
30
kDa

Figure S4

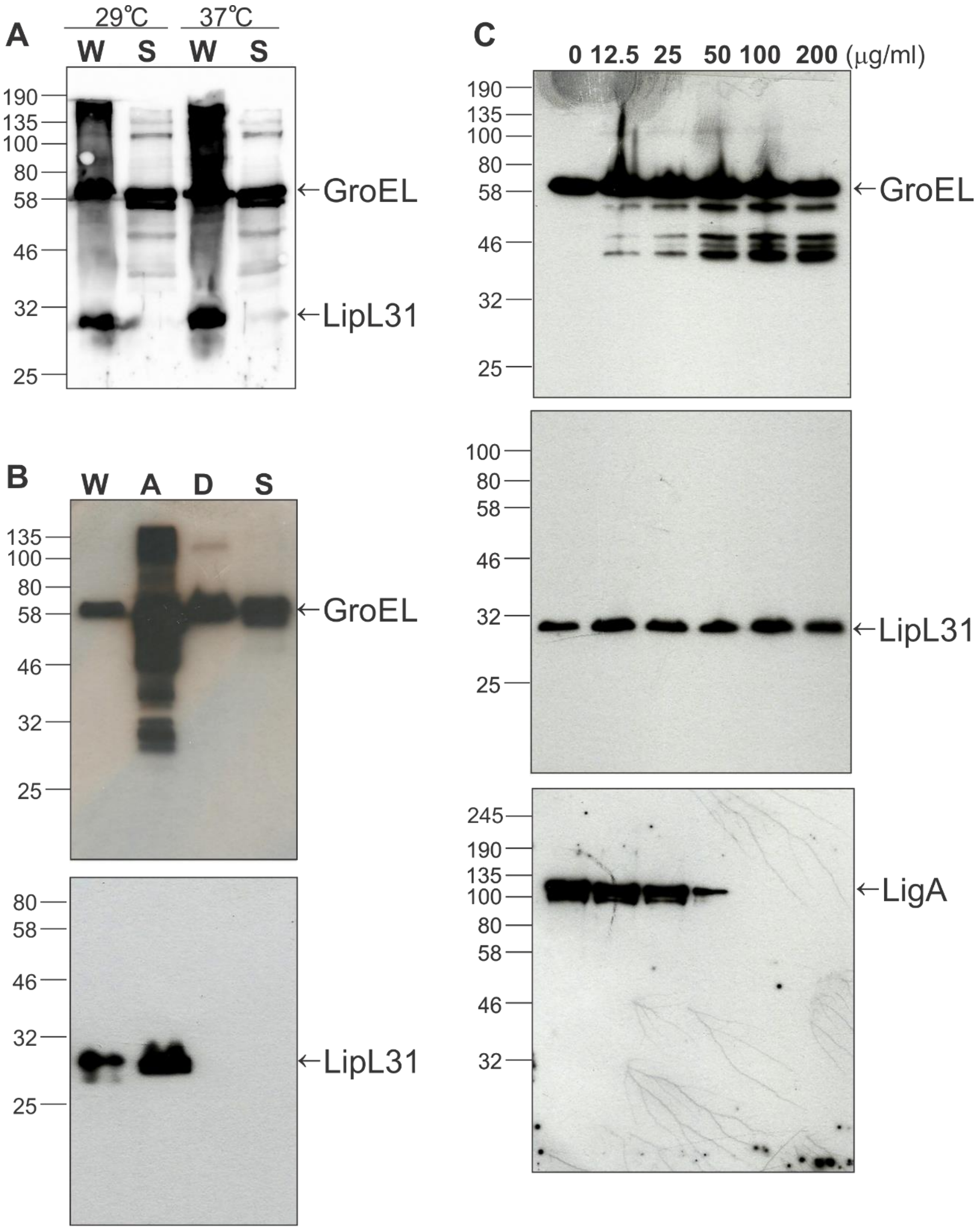
A
29°C
37°C
W
S
W
S
190
135
100
80
58
46
32
25
GroEL
LipL31
B
W
A
D
S
135
100
80
58
46
32
25
GroEL
80
58
46
32
25
LipL31
C
0 12.5 25 50 100 200 (μg/ml)
190
135
100
80
58
46
32
25
GroEL
100
80
58
46
32
25
LipL31
245
190
135
100
80
58
46
32
LigA

Figure S5

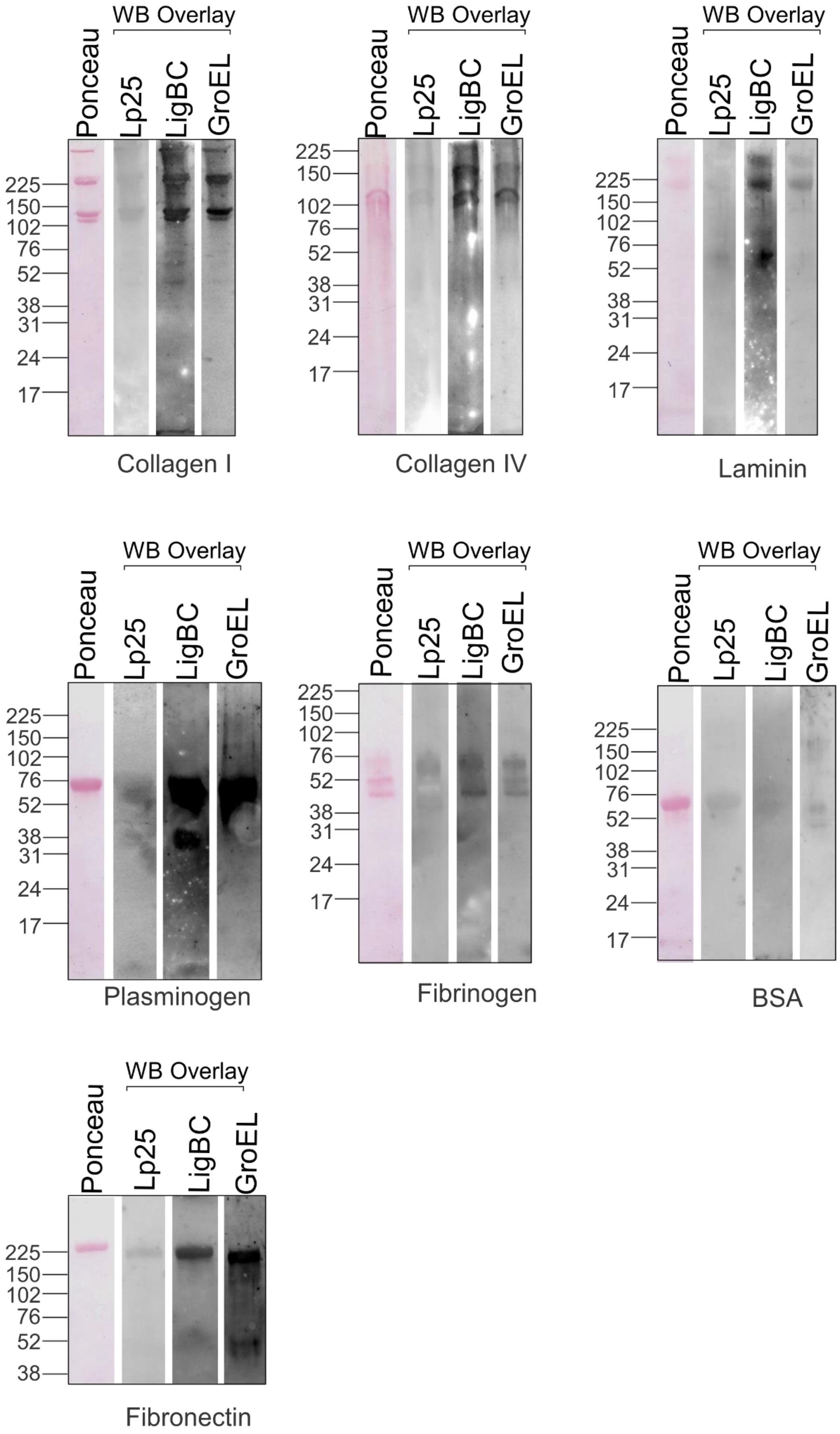
WB Overlay
Ponceau
Lp25
LigBC
GroEL
225
150
102
76
52
38
31
24
17
Collagen I
Collagen IV
Laminin
Plasminogen
Fibrinogen
BSA
Fibronectin
